# ICOS‐ImmunoPET Monitors T Cell Activation After Combination Immune Checkpoint Blockade in a Mouse Model of Melanoma Brain Metastasis

**DOI:** 10.1002/advs.76803

**Published:** 2026-08-15

**Authors:** Renesmee C. Kuo, Rohit Verma, Sydney C. Nagy, Mausam Kalita, Samuel Aubert, Josué Ballesteros‐Álvarez, Pedro Machado Almeida, Hannes Vogel, Israt S. Alam, Michael Lim, Michelle L. James

**Affiliations:** ^1^ Department of Electrical Engineering Stanford University Stanford California USA; ^2^ Molecular Imaging Program at Stanford Department of Radiology Stanford University Stanford California USA; ^3^ Department of Neurosurgery Stanford University Stanford California USA; ^4^ Lunaphore Technologies Tolochenaz Switzerland; ^5^ Department of Pathology Stanford University Stanford California USA; ^6^ Department of Neurology and Neurological Sciences Stanford University Stanford California USA

**Keywords:** brain metastases, immune checkpoint blockade, immunoPET, melanoma, T cell activation

## Abstract

Immune checkpoint inhibitors (ICIs) have transformed outcomes for melanoma brain metastases (MBM), yet early response assessment remains challenging because currently available tools such as magnetic resonance imaging (MRI) lack molecular specificity and are confounded by pseudoprogression. Antibody‐based positron emission tomography (immunoPET) is a highly sensitive and specific molecular imaging technique that enables noninvasive, whole‐body visualization of immune dynamics in vivo. The inducible T cell costimulatory receptor (ICOS) is a promising biomarker of therapy‐induced T cell activation. Here, a previously validated tracer, [^89^Zr]DFO‐ICOS mAb, is used to track activated T cells in a murine MBM model treated with combined anti‐PD‐1/anti‐CTLA‐4 therapy, and immunoPET signal is shown to correspond with therapeutic response and intratumoral T cell activation. To establish clinical relevance of ICOS‐immunoPET, single‐cell RNA sequencing data from 6 human MBM specimens are reanalyzed, revealing significantly higher ICOS expression accompanied by coordinated activation signatures in ICI‐treated tumors. These findings support ICOS‐immunoPET as a promising strategy for earlier, noninvasive, functionally informative assessment of immunotherapy response, with potential to improve patient stratification in brain metastases.

## Graphical abstract

ICOS‐immunoPET noninvasively visualizes activated T cells in melanoma brain metastases following combined anti‐PD‐1/anti‐CTLA‐4 therapy. Clinical translation of this imaging approach may improve patient stratification and treatment monitoring and serve as a quantitative imaging endpoint to support immunotherapy development and clinical trials in brain metastases.

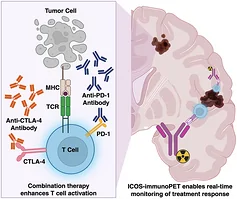

## 1 Introduction

Among solid tumors, melanoma has one of the highest propensities to metastasize to the brain, occurring in up to 40%–60% of patients [1]. Despite advances in therapy, the prognosis for melanoma brain metastases (MBM) remains poor, with median survival typically less than one year [2, 3]. Immune checkpoint inhibitors (ICI) have transformed the therapeutic landscape by blocking inhibitory signals that restrain T cell activation, thereby restoring antitumor immune responses [4]. Combination regimens targeting programmed death 1 (PD‐1) and cytotoxic T lymphocyte‐associated protein 4 (CTLA‐4) release complementary brakes on T cell priming and effector function, resulting in enhanced intracranial activity, more potent systemic responses, and more durable tumor control compared to monotherapy [5]. However, due to disease heterogeneity and complex tumor‐immune escape mechanisms, resistance to ICIs is common, and a substantial proportion of patients exhibit incomplete or absent responses [6]. Consequently, there is an urgent need for noninvasive tools capable of assessing in vivo immune dynamics associated with therapeutic success or failure.

Current clinical response evaluation for brain tumors relies on serial magnetic resonance imaging (MRI) interpreted using the Response Assessment in Neuro‐Oncology (RANO) criteria [7]. While MRI is the standard for visualizing anatomic and structural changes, it fails to provide insight into the underlying molecular responses that precede structural changes. In the immuno‐oncology setting, MRI is further confounded by pseudoprogression—an early radiographic worsening driven not by tumor growth but by immune cell infiltration, inflammation, and transient blood‐brain barrier (BBB) disruption [8]. As a result, reliable response determination often cannot be made until 9–12 weeks after treatment initiation [9], delaying critical clinical therapeutic decisions and obscuring early signals of response or resistance.

Positron emission tomography (PET) offers a promising complementary, noninvasive strategy to dynamically quantify molecular responses across the whole body. The most widely used tracer for oncology applications, [^18^F]FDG, measures glucose metabolism but has limited utility in neuro‐oncology due to high baseline uptake in cerebral gray matter and poor tumor‐to‐background contrast [10, 11]. Amino acid tracers such as [^18^F]FET and [^18^F]FDOPA improve lesion conspicuity in the brain, yet their uptake predominantly reflects LAT1‐mediated amino acid transport and provides limited specificity for immune activation [12]. Small molecule immune PET tracers, including nucleoside analogues such as [^18^F]F‐AraG and [^18^F]CFA, enable visualization of T cell activation through the nucleoside salvage pathway, but their specificity is constrained by shared metabolic pathways in other proliferating or inflamed tissues [13, 14].

ImmunoPET approaches using monoclonal antibodies (mAb) against immune cell surface markers can overcome these limitations by enabling visualization of specific immune cell populations in vivo [15]. Tracers targeting CD3 [16], CD4 [17], CD7 [18], or CD8 [19] can quantify the presence and spatial distribution of T cell populations within tumors, but they do not directly capture the functional state of T cells. As T cell activation and expansion of antigen‐specific T cells is requisite for effective immune checkpoint blockade, early response prediction necessitates biomarkers that specifically capture immune activation. Activation‐associated markers such as OX40 [20, 21], HLA‐DR [22], and CD69 [23, 24] have shown promise in preclinical studies as dynamic indicators of T cell response to different classes of cancer immunotherapies.

In this work, we address the critical need for an imaging biomarker capable of detecting early T cell activation within MBM during immune checkpoint blockade. The inducible T cell costimulatory receptor (ICOS), a member of the CD28/CTLA‐4 family, is rapidly and robustly upregulated upon T cell activation [25]. Prior studies demonstrate that ICOS expression is induced early after PD‐1 and CTLA‐4 blockade and exhibits sustained expression kinetics [26, 27], making it well suited to capture the dynamic immune responses elicited by ICIs. ICOS upregulation consistently marks activated, therapy‐engaged effector T cells and has been associated with antitumor activity. Our previous work describes the development of the first PET tracer targeting ICOS,  [^89^Zr]DFO‐ICOS mAb, and its rigorous validation regarding its specificity for detecting ICOS‐expressing murine activated T cells in models of lung cancer [25], as well as systemic models such as graft‐versus‐host disease [28] and B‐cell lymphoma [29].

Leveraging our previously validated tracer, here we evaluate ICOS‐immunoPET for the first time in the context of a mouse model of MBM as an early, noninvasive predictor of therapeutic response to combination PD‐1 and CTLA‐4 blockade. To provide orthogonal validation of PET findings and deeper biological context, we further characterize the spatial distribution and activation state of intratumoral T cells using multiplexed spatial proteomics in therapy‐ and vehicle‐treated murine MBM. To establish translational relevance, we reanalyze a publicly available single‐cell RNA sequencing (scRNA‐seq) dataset from MBM patient samples treated with or without ICIs to further define ICOS‐associated T cell activation signatures in human disease. Together, these complementary in vivo imaging, spatial proteomics in murine brain metastasis, and transcriptomic analyses of human brain metastases aim to establish ICOS‐immunoPET as a clinically translatable tool for probing immune activation and guiding immunotherapy decision‐making in neuro‐oncology.

## 2 Results

### 2.1 ICOS Expression is Upregulated in Human Melanoma Brain Metastases After Immune Checkpoint Inhibitor Therapy

To assess the clinical relevance of ICOS as a potential biomarker of immune activation following immune checkpoint blockade, we reanalyzed a publicly available scRNA‐seq dataset from MBM patient samples treated with or without ICI (GSE193745) [30]. MBM samples included in the ICI responder group were selected from patients who demonstrated survival benefit following ICI therapy. Given the central role of immune checkpoint blockade in reshaping the tumor immune microenvironment, we sought to determine whether ICOS expression is associated with therapy‐induced immune activation in human MBM.

Unsupervised Leiden clustering identified 13 major cell clusters in no‐treatment samples and 17 clusters in ICI‐treated samples, which were visualized using t‐distributed stochastic neighbor embedding (t‐SNE) plots (Figure 1A). Cell types were annotated based on established marker gene signatures reported in previous studies, revealing seven major cellular lineages across both conditions. Tumor cells, characterized by high expression of epithelial markers such as *KRT8* and *EPCAM*, were predominantly represented in clusters 7 and 8 in no‐treatment samples and clusters 10, 11, and 13 following ICI treatment. Endothelial cells and pericytes, identified by canonical BBB‐associated genes including *VWF, CLDN5, CDH5*, and *ESAM*, were observed in clusters 2 and 6 in the ICI responder samples. Myeloid and macrophage populations were annotated based on robust expression of *MRC1, CD163, TREM2*, and *MSR1*, corresponding to clusters 3 and 6 in no‐treatment samples and clusters 7, 8, and 15 after ICI therapy. T cell populations, defined by expression of canonical T cell markers (*CD3, CD28, CD2, TRBC1, TRAC*, and *SKAP1*), were identified in cluster 5 in no‐treatment samples and expanded across clusters 1, 4, 5, and 9 in ICI‐treated samples. Oligodendrocytes, marked by expression of *MBP, PLP1, MOG*, and *MAG*, were observed in clusters 0 and 12 in the ICI responder group. In contrast, clusters 0 and 12 in no‐treatment samples were classified as plasma cells based on high expression of *MZB1* and *JCHAIN*. Fibroblast populations, characterized by expression of extracellular matrix genes including *COL1A1* and *COL1A2*, were identified in cluster 10 in no‐treatment samples and clusters 3, 14, and 16 in ICI‐treated samples. Several clusters in the no‐treatment cohort (clusters 1, 2, 4, 9, and 11) lacked clear enrichment for known lineage‐specific markers and were therefore annotated as unknown.

**FIGURE 1:**
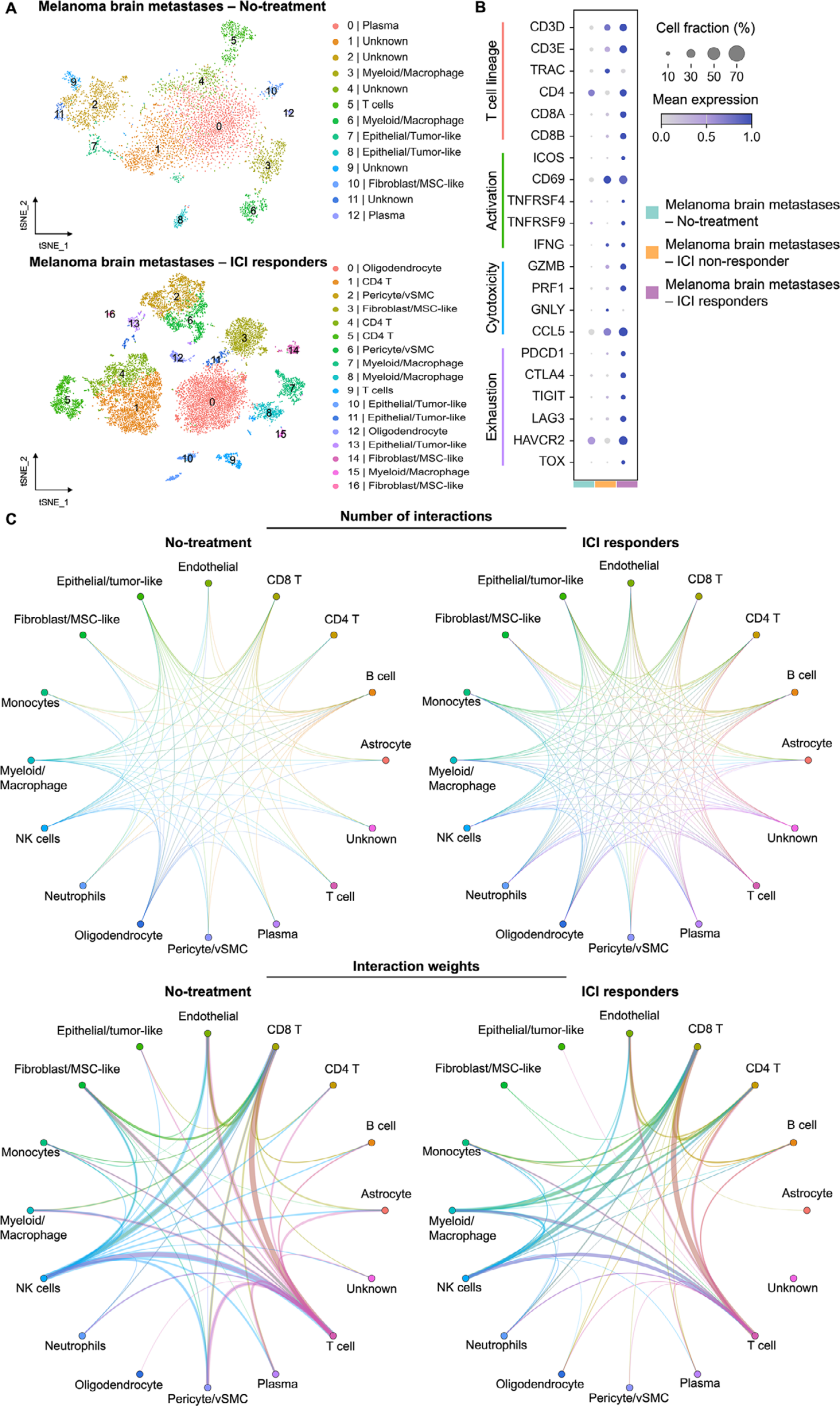
Immune checkpoint blockade remodels immune landscape and enhances intercellular communication in MBM. (A) t‐SNE visualization of scRNA‐seq data from MBM patient samples with (top) and without ICI (bottom). Cells are colored by unsupervised Leiden clusters and annotated based on established lineage‐specific marker genes. (B) Dot plot showing the relative expression and percentage of T cell lineage, activation, cytotoxicity, and exhaustion‐associated genes across MBM samples from no‐treatment, ICI non‐responder, and ICI responder patients. Dot size represents the fraction of cells expressing each gene, and color intensity indicates scaled mean expression. (C) Circular network plots summarizing cell‐cell communication inferred from pathway‐based expression analysis. Top panels show the number of interactions and bottom panels show interaction weights (summed interaction strength) for no‐treatment and ICI responder samples. Nodes represent major cell types, while edges depict inferred signaling interactions, with line thickness corresponding to interaction magnitude and line color indicating the originating (sender) cell type.

To further characterize immune activation induced by ICI therapy, we examined the expression of T cell lineage, activation, cytotoxicity, and checkpoint‐associated genes across MBM samples from no‐treatment, ICI responder, and ICI non‐responder cohorts (Figure 1B). Relative to untreated tumors, both ICI responder and non‐responder samples demonstrated increased expression of canonical T cell markers, including *CD3D*, *CD3E*, *TRAC*, *CD4*, *CD8A*, and *CD8B*, indicating enhanced T cell infiltration following checkpoint blockade. Notably, *ICOS* expression was largely absent in untreated and ICI non‐responder tumors, whereas ICI responder samples exhibited a marked enrichment of *ICOS*‐expressing T cells. Mean *ICOS* expression was increased 2.04‐fold in responder tumors relative to non‐responders, compared with a more modest 1.37‐fold increase in *CD8A* and *CD8B* expression, suggesting that ICOS more strongly distinguishes responses to immune checkpoint blockade than T cell lineage markers alone. Consistent with this observation, increased *ICOS* expression was accompanied by elevated expression of activation‐associated markers, including *CD69*, *TNFRSF4* (OX40), *TNFRSF9* (4‐1BB), and *IFNG*, supporting that ICOS identifies a highly activated T cell state associated with clinical response to checkpoint blockade. Similarly, cytotoxic effector genes, including *GZMB*, *PRF1*, *GNLY*, and *CCL5*, were increased following ICI treatment, with responder tumors demonstrating the strongest cytotoxic signature. Responder samples also exhibited a more coordinated immune activation phenotype characterized by concurrent expression of activation, cytotoxicity, and checkpoint‐associated genes, whereas non‐responder tumors displayed evidence of T cell infiltration without a comparable activation state. Consistent with this observation, checkpoint and exhaustion‐associated markers, including *PDCD1*, *CTLA4*, *TIGIT*, *LAG3*, *HAVCR2*, and *TOX*, were elevated in responder tumors, reflecting the accumulation of chronically stimulated tumor‐reactive T cells within the tumor microenvironment (TME). Taken together, these findings identify ICOS as a clinically relevant biomarker of immunotherapy response in MBM, reflecting the presence of activated tumor‐reactive T cells and providing a rationale for ICOS‐targeted molecular imaging.

Given that immune activation is not solely defined by cell‐intrinsic transcriptional programs but also by dynamic intercellular signaling, we next assessed how ICI alters cell‐cell communication networks within the MBM microenvironment (Figure 1C). Using an expression‐based ligand‐receptor interaction framework, we quantified both the number of inferred interactions and their aggregate interaction weights between major immune and stromal cell types. In no‐treatment samples, cell‐cell communication was comparatively sparse, with limited interaction density and lower overall signaling strength across immune compartments. In contrast, ICI responder samples displayed a pronounced increase in both the number and strength of inferred interactions, particularly involving T cells, myeloid/macrophage populations, and endothelial and stromal compartments. Enhanced interactions were observed between CD4^+^ and CD8^+^ T cells and antigen‐presenting myeloid populations, consistent with heightened immune engagement and coordination following checkpoint blockade. Importantly, interaction weights involving T cell subsets were markedly increased in ICI responder samples, indicating not only a greater diversity of signaling interactions but also stronger cumulative signaling intensity.

### 2.2 Preclinical Modeling Recapitulates Therapy‐Induced ICOS Upregulation Observed in Human MBM

Having established that ICOS is upregulated in human MBM following immune checkpoint blockade, we next sought to determine whether this biology could be recapitulated in a preclinical model suitable for immunoPET tracer evaluation. To this end, we adapted and optimized a murine B16 melanoma model previously described by Taggart et al. [31] that incorporates both intracranial and extracranial disease, thereby recapitulating a clinically relevant setting of metastatic melanoma with brain involvement (Figure 2A). Briefly, B16F1‐luc melanoma cells were implanted subcutaneously into the right flank, followed two days later by intracranial implantation to establish MBM. Brain tumor establishment was confirmed on by bioluminescence imaging (BLI) four days after intracranial implantation, and mice with either minimal or excessively large tumors were excluded to minimize variability between experimental groups. Combination anti‐PD‐1/anti‐CTLA‐4 therapy has previously demonstrated efficacy in a similar mouse model, with response rates comparable to those observed clinically in patients with MBM [32, 33].

**FIGURE 2:**
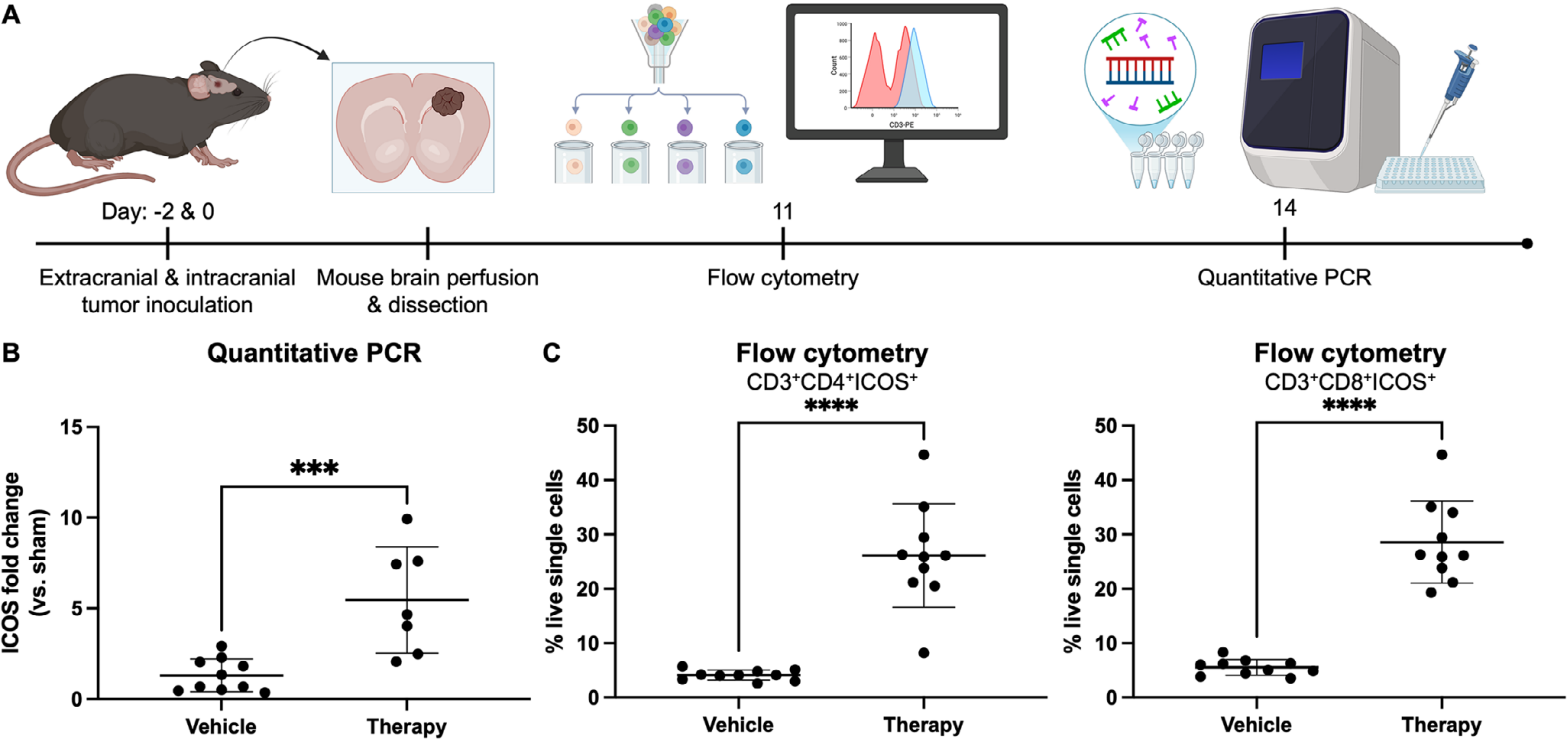
Immune checkpoint blockade increases trafficking of ICOS^+^ activated T cells to brain tumors in a melanoma brain metastasis mouse model. (A) Schematic of the experimental design. C57BL/6J mice were implanted with extracranial B16F1‐luc melanoma tumors in the right flank, followed two days later by intracranial implantation to establish MBM. Whole brains were harvested for flow cytometry and qPCR analyses on days 11 and 14 post‐intracranial tumor implantation, respectively. (B) qPCR analysis of Icos expression in whole‐brain tissue from vehicle‐treated (*n* = 10) and therapy‐treated (*n* = 7) MBM mice, shown as fold change relative to sham‐operated controls. (C) Percentage of CD3^+^ CD4^+^ICOS^+^ and CD3^+^CD8^+^ICOS^+^ T cells in whole‐brain samples from vehicle‐ and therapy‐treated MBM mice (*n* = 10/group), as determined by flow cytometry. Statistical analysis was performed using the Mann‐Whitney test. * denotes direct comparison of groups (^***^ *p* < 0.001, ^****^ *p* < 0.0001). Data are mean ± SD.

Using this optimized model, we assessed whether ICOS expression is induced in T cells within the brain tumor microenvironment following immune checkpoint blockade at both the transcriptomic and proteomic levels. Quantitative polymerase chain reaction (qPCR) analysis of whole brain tissue collected on day 14 post‐intracranial tumor implantation demonstrated a significant upregulation of *Icos* transcripts in mice treated with combined anti‐PD‐1/anti‐CTLA‐4 therapy (100 µg/100 µL each, via intraperitoneal [i.p.] injection) compared with vehicle‐treated (saline, i.p.) controls, corresponding to a 4.2‐fold increase in *Icos* expression (Figure 2B; p = 0.0007). Consistent with these transcriptional changes, flow cytometric analysis performed on day 11 post‐implantation revealed a marked increase in ICOS^+^CD4^+^ and ICOS^+^CD8^+^ T cells in the whole brain of therapy‐treated mice relative to vehicle‐treated controls (6.36‐fold and 5.15‐fold, respectively; *p* <0.0001; Figure 2C and Figure S1).

### 2.3 ICOS‐immunoPET Enables Visualization of Activated T Cells Following Anti‐PD‐1/Anti‐CTLA‐4 Treatment in Murine Melanoma Brain Metastasis

Following confirmation of therapy‐induced ICOS upregulation in the murine MBM model with both extracranial flank tumors and intracranial brain tumors, we next evaluated whether ICOS‐immunoPET could noninvasively visualize activated T cell responses following immune checkpoint blockade (Figure 3A). Following initiation of combination therapy on days 5, 7, and 9 post‐intracranial tumor inoculation, extracranial tumor growth began to plateau by day 9, which coincided with the last day of treatment administration. A significant difference in extracranial tumor volume between therapy‐treated and vehicle‐treated mice was observed from day 12 onward (p<0.0001; Figure 3B). Intracranial tumor burden, quantified by T2‐weighted (T2w) MRI on day 14 post‐intracranial tumor inoculation, was also significantly reduced in the therapy‐treated group compared with vehicle controls (55.06 vs. 116.13 mm^3^; p = 0.03; Figure S2). Correspondingly, mice receiving combination immunotherapy exhibited significantly prolonged survival relative to vehicle‐treated controls (p<0.01; Figure 3C), confirming robust therapeutic efficacy in this model.

**FIGURE 3:**
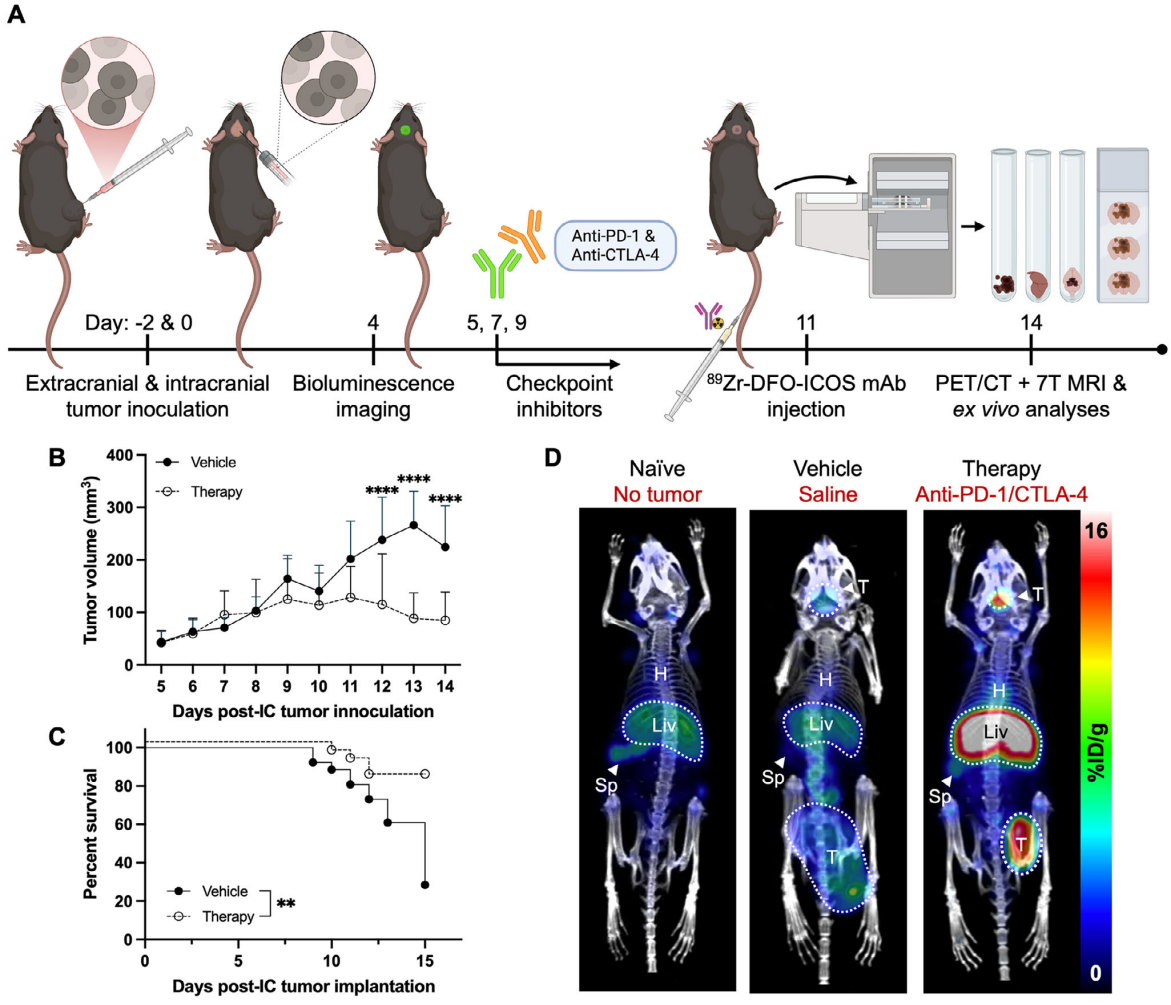
ICOS‐immunoPET enables noninvasive visualization of activated T cells following immune checkpoint blockade in a murine melanoma brain metastasis model. (A) Study design and imaging timeline for ^89^Zr‐DFO‐ICOS mAb PET/CT/MR imaging in mice bearing intracranial and extracranial melanoma, treated with combination ICI therapy or vehicle. All mice were implanted with an extracranial tumor on the right flank on day ‐2, followed by intracranial tumor implantation in all mice on day 0. BLI was performed on day 4 post‐intracranial tumor implantation to confirm brain tumor presence. A group of mice received combination checkpoint therapy anti‐PD‐1/CTLA‐4 on days 5, 7, and 9, and all mice were injected with ^89^Zr‐DFO‐ICOS mAb followed by PET/CT/MR imaging and ex vivo analyses on day 14. (B) Extracranial tumor growth curves (*n* = 14 vehicle‐treated and *n* = 13 therapy‐treated mice) and (C) overall survival of MBM mice treated with vehicle (*n* = 26) or combination ICI therapy (*n* = 24). Mice were randomized to treatment or vehicle groups on day 5 following intracranial tumor implantation and received therapy on days 5, 7, and 9. (D) Representative whole‐body maximum intensity projection (MIP) ^89^Zr‐DFO‐ICOS mAb PET/CT images of naïve and MBM mice with both intracranial and extracranial tumors 72 h post‐injection of tracer. For visualization purposes, the survival curve of the treatment group in (C) was vertically offset by three data units to avoid overlap with the vehicle group. Statistical analyses were performed using (B) two‐way ANOVA with Tukey's multiple comparisons test or (C) log‐rank test. * denotes direct comparison of groups (^**^ *p*<0.01, ^****^ *p*<0.0001). Data are mean + SD.

Having established treatment response, we evaluated the ability of ICOS‐immunoPET to visualize activated T cells in vivo using ^89^Zr‐DFO‐ICOS mAb. Deferoxamine (DFO) conjugation conditions were optimized and confirmed via electrospray ionization mass spectrometry, which demonstrated major peaks corresponding to unconjugated ICOS mAb (*m/z* 149935.2) and DFO‐conjugated species with mAb‐to‐DFO ratios within the desired range of 1:1 (*m/z* 150686.0) and 1:2 (*m/z* 151438.0; Figure S3A,B). The DFO‐ICOS mAb was subsequently radiolabeled with ^89^Zr‐oxlate with high reproducibility, yielding ^89^Zr‐DFO‐ICOS mAb radiotracer with a radiolabeling purity of 95.98% as determined by radio‐instant thin‐layer chromatography (iTLC; *n* = 3), and a specific activity of 8.32 µCi/µg (or 0.308 MBq/µg; Figure S3C and Table S1).

Following optimized radiolabeling, the ^89^Zr‐DFO‐ICOS mAb radiotracer was administered intravenously on day 11 post‐intracranial tumor implantation, and PET/computed tomography (CT; 20‐min static scans) and MRI was performed 72 h after injection for optimal signal‐to‐background contrast. Imaging was performed in naïve, vehicle‐treated, and therapy‐treated MBM‐bearing mice. In naïve mice, ^89^Zr‐DFO‐ICOS mAb primarily accumulated in the heart, spleen, and liver, with minimal bone uptake, reflecting circulating antibody kinetics and hepatic clearance, consistent with previously published studies on this immunoPET tracer (Figure 3D) (25,28,29). In the vehicle‐treated tumor‐bearing mice, tracer distribution closely resembled that observed in naïve animals, with uptake largely confined to highly perfused organs; moderate PET signal was observed in the intracranial and extracranial tumor regions. In contrast, MBM‐bearing mice treated with combined anti‐PD‐1/anti‐CLTA‐4 therapy exhibited markedly increased ^89^Zr‐DFO‐ICOS mAb accumulation within both brain and flank tumors compared with vehicle controls (brain tumors: 4.41 vs. 3.28%ID/g [percent injected dose per gram], flank tumors: 7.07 vs. 4.99%ID/g; p<0.01; Figures 3D and 4A,B). Quantitative PET analysis further demonstrated enhanced tumor‐to‐background contrast in therapy‐treated animals, with higher intracranial tumor‐to‐healthy brain tissue ratios (4.74 vs. 3.95) and extracranial tumor‐to‐muscle ratios (12.63 vs. 9.42) relative to vehicle‐treated mice (Figure 4C and Table S2).

**FIGURE 4:**
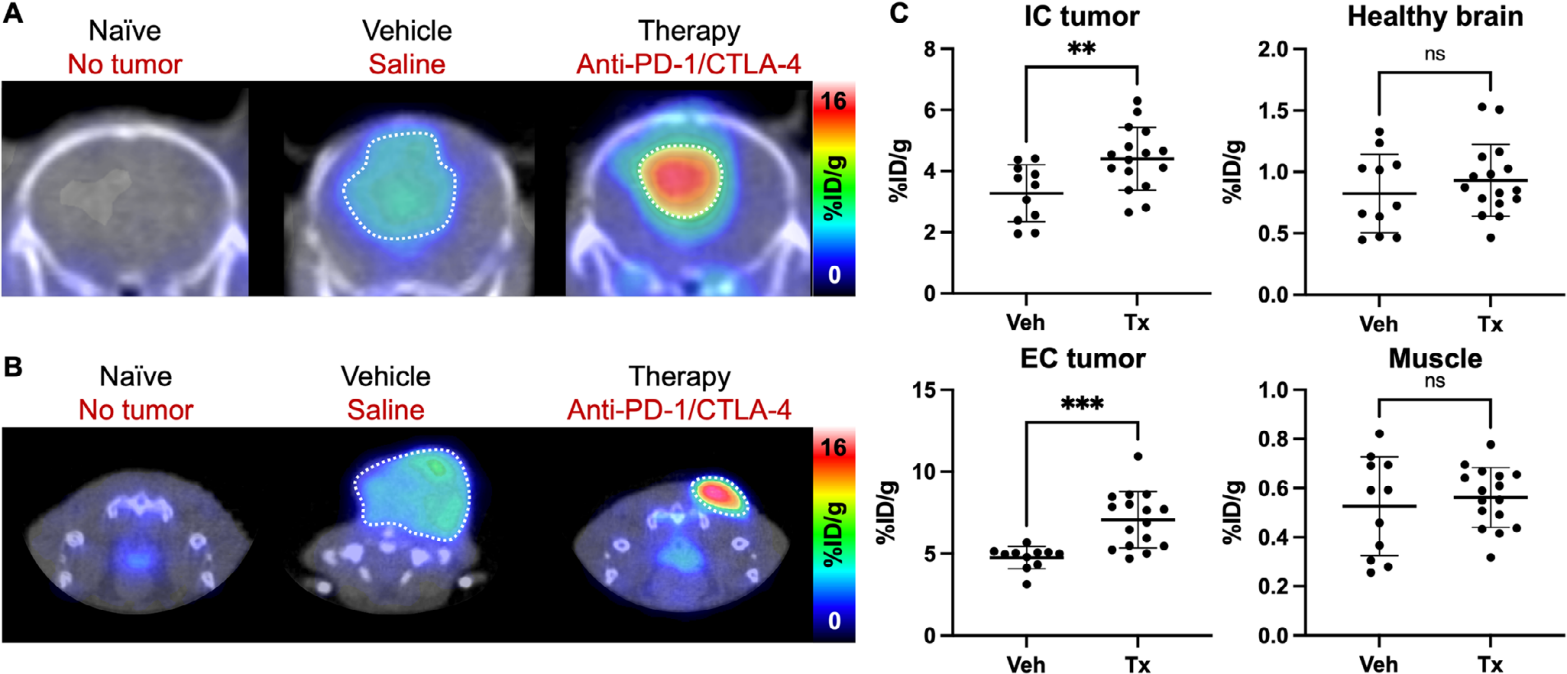
In vivo ICOS‐immunoPET imaging enables visualization of ICOS^+^ T cell trafficking to intracranial and extracranial tumors in brain metastasis mice. (A) Representative ICOS‐immunoPET/CT images highlighting elevated PET signal in intracranial tumors and (B) extracranial tumors (white dotted lines) in vehicle‐ and therapy‐treated mice and naïve controls. (C) Quantification of ^89^Zr‐DFO‐ICOS mAb PET images in intracranial tumors, healthy brain region, extracranial tumors, and contralateral healthy muscle in MBM mice. *n* = 11 vehicle‐ and *n* = 16 therapy‐treated mice. Statistical analysis was performed using Mann‐Whitney tests. * denotes direct comparison of groups (ns: P>0.05, ^**^ *p*<0.01, ^***^ *p*<0.001). IC = intracranial; EC = extracranial; Veh = vehicle; Tx = therapy. Data are mean ± SD.

To corroborate in vivo PET imaging findings, mice were perfused to remove the possible contribution of tracer in the blood pool following the 72 h PET/CT/MR acquisition, and high‐resolution ex vivo autoradiography and gamma counting were subsequently performed. Gamma counting confirmed significantly higher ^89^Zr‐DFO‐ICOS mAb binding in whole brain tissue and lymphoid organs of therapy‐treated mice compared with vehicle‐treated controls (p<0.05; Figure 5). In contrast, tracer signal in blood was significantly lower in therapy‐treated mice relative to vehicle controls (p<0.0001), likely due to reduced amount of circulating free tracer resulting from enhanced sequestration within tumors and lymphoid tissues. Ex vivo autoradiography of 40 µm brain sections further validated the PET findings, demonstrating elevated radiotracer binding localized to the intracranial tumor regions in therapy‐treated mice, with quantitative analysis closely mirroring in vivo PET and gamma counting results (Figure 6).

**FIGURE 5:**
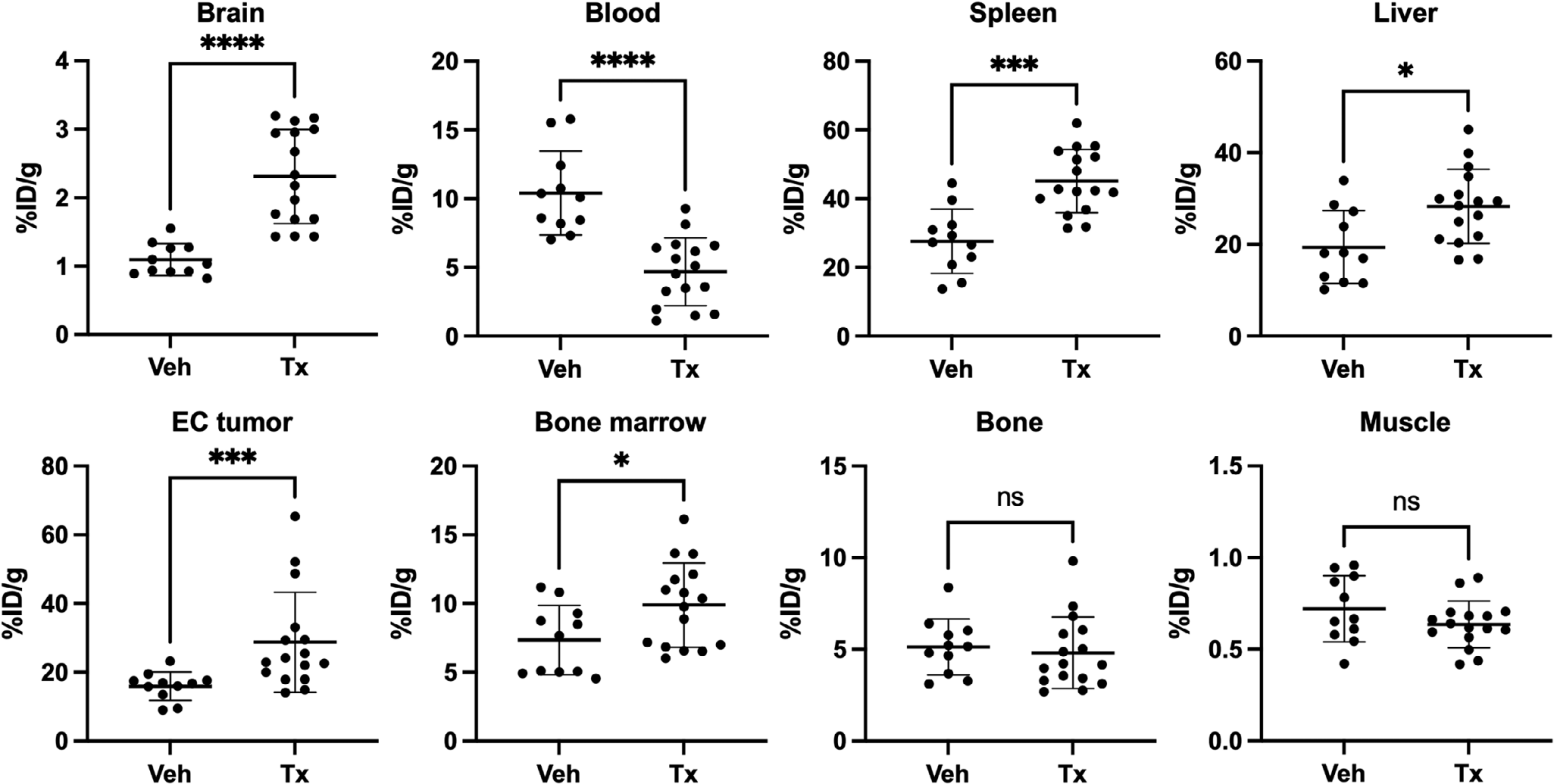
Quantitative biodistribution analysis of ^89^Zr‐DFO‐ICOS mAb corroborates PET imaging findings. Vehicle (*n* = 11) and therapy‐treated (*n* = 16) mice were euthanized for ex vivo gamma counting of tissues following PET imaging 72 h after tracer injection. Statistical analysis was performed using Mann‐Whitney tests. * denotes direct comparison of groups (ns: *p*>0.05, ^*^ *p*<0.05, ^**^ *p*<0.01, ^***^ *p*<0.001, ^****^ *p*<0.0001). Veh = vehicle; Tx = therapy. Data are mean ± SD.

**FIGURE 6:**
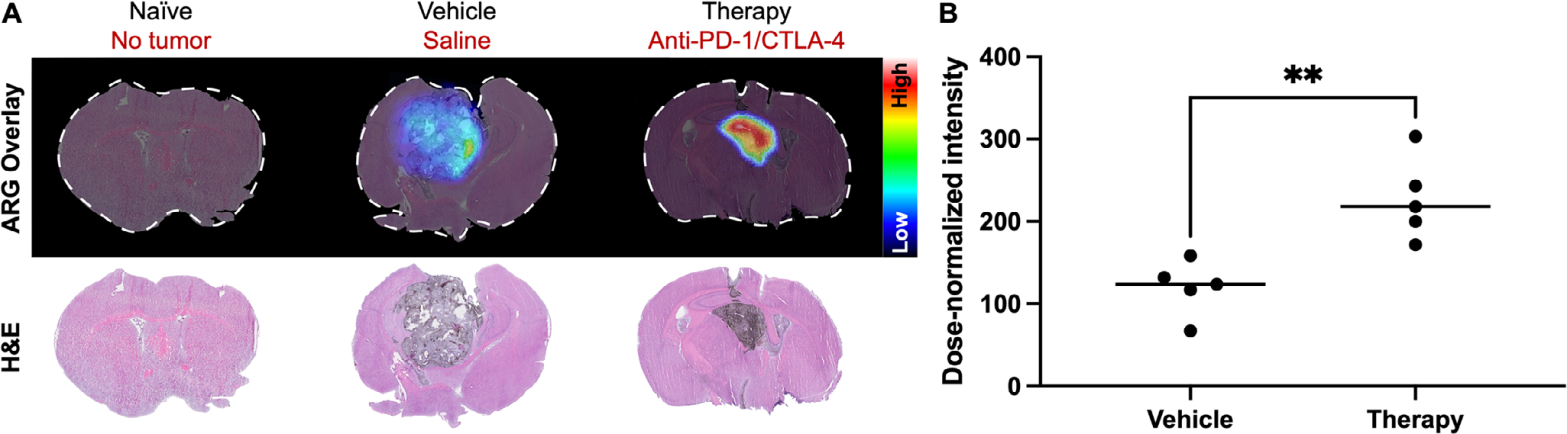
Ex vivo autoradiography provides high‐resolution visualization of ^89^Zr‐DFO‐ICOS mAb binding to ICOS^+^ activated T cells. (A) Representative ex vivo autoradiography images of the whole brains from naïve, vehicle‐, and therapy‐treated brain metastasis mice. (B) Quantification of autoradiography signal from the brain tumor ROI of vehicle‐ (*n* = 5) and therapy‐treated mice (*n* = 5). Statistical analysis was performed using the Mann‐Whitney test. * denotes direct comparison of groups (^**^ *p*<0.01). Data are mean ± SD.

Notably, PET imaging and gamma counting also revealed significantly increased tracer binding in the liver of therapy‐treated mice compared with vehicle controls. Subsequent flow cytometric analysis confirmed marked infiltration of ICOS^+^CD8^+^ T cells in the livers of treated animals (2.31‐fold increase; *p*<0.01), with a trend toward significance observed in ICOS^+^CD4^+^ T cells (1.16‐fold; p = 0.16), concordant with PET quantification of the liver (1.46‐fold increase; p = 0.0018; Figure S4). As antibodies are primarily metabolized and cleared through the hepatobiliary system, the lower observed fold change is likely due to circulating tracer and hepatic catabolism. This pattern of increased hepatic tracer binding indicates the presence of liver‐mediated immune injury, a well‐documented immune‐related adverse event associated with combination PD‐1/CTLA‐4 immunotherapy [34], and further demonstrates that ICOS‐immunoPET enables noninvasive visualization of both on‐tumor immune activation and systemic immune engagement following checkpoint blockade.

### 2.4 Multiplexed Immunofluorescent Imaging Reveals Spatial Reorganization of the Murine Intracranial Tumor Microenvironment Following Checkpoint Blockade

To elucidate the cellular mechanism underlying therapeutic response in this murine MBM model and to assess the spatial overlap between ICOS‐immunoPET signal and immune activation, we leveraged multiplexed spatial profiling using sequential immunofluorescence (seqIF) on the COMET platform [35]. This approach enables high‐dimensional protein visualization at single‐cell resolution and direct interrogation of cell‐cell interactions within intact tissue. Our antibody panel was designed to identify major immune populations, including lymphoid cells (T and B cells), myeloid‐lineage cells (microglia, tumor‐associated myeloid cells, and dendritic cells), and non‐immune cells such as endothelial cells, astrocytes, and neurons (Table S3). We processed COMET images using channel‐specific pixel‐wise background subtraction with normalized subtraction, followed by nuclei‐based cell segmentation using a StarDist‐based machine learning model [36, 37] to generate single‐cell annotations (Figure S6A). Rule‐based phenotyping was then applied to classify individual cells into defined populations, and cell counts were quantified from representative whole‐brain tissue sections from vehicle‐ and therapy‐treated mice determined from PET imaging and biodistribution analyses (Figure 7A and Figure S6B).

**FIGURE 7:**
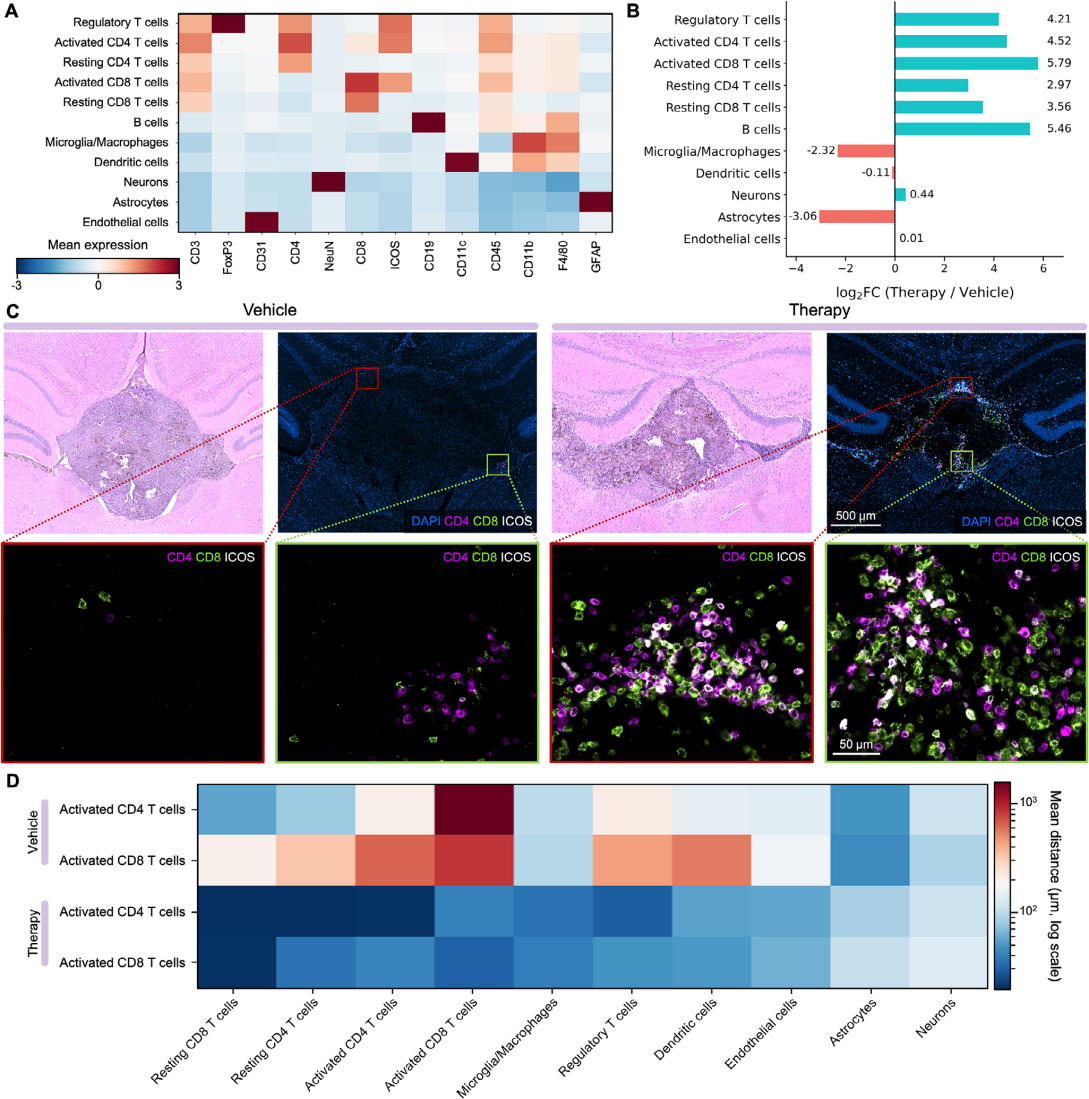
Combination immune checkpoint blockade enhances T cell infiltration and activation in the brain tumor microenvironment of melanoma brain metastases. (A) Visual representation of a z‐normalized expression matrix of major immune, neuronal, and endothelial cell classes in a whole brain section of tumor‐bearing mice. (B) Log_2_ fold‐change (log_2_FC) in cellular abundance between representative therapy‐treated and vehicle‐treated brain sections, calculated as Therapy/Vehicle for each phenotypic class. Positive values (turquoise) indicate enrichment following therapy, whereas negative values (coral) indicate reduced abundance. (C) Representative whole‐brain tissue sections from vehicle‐ and therapy‐treated mice. Top: H&E and multiplex immunofluorescence showing DAPI (blue, nuclei), CD4 (pink, helper T cells), CD8 (green, cytotoxic T cells), and ICOS (white, T cell activation marker). Bottom: High‐magnification views of tumor border regions showing activated T cell infiltration in mice with and without therapy. (D) T cell‐centric neighborhood analysis showing mean distance (µm; log scale) between activated CD4^+^ and activated CD8^+^ T cells and neighboring cell populations across the tumor‐bearing brain region.

Compared with vehicle controls, representative therapy‐treated brain sections exhibited increased infiltration of T cells, including regulatory T cells and resting and activated CD4^+^ and CD8^+^ T cells, accompanied by a reduction in tumor‐associated myeloid cells (TAMCs) and reactive astrocytes (Figure 7B and Figure S6C). While these analyses were performed on representative animals and were not intended as a quantitative cohort‐level assessment, the observed differences in cellular composition provide supportive spatial evidence of enhanced immune infiltration following therapy. Importantly, these findings mirror the increased ICOS^+^ T cell populations shown by flow cytometry and are consistent with the elevated ICOS‐immunoPET and autoradiography signal observed in therapy‐treated animals, providing spatial confirmation of the therapy‐induced immune response within the brain TME. Spatial analysis focused on tumor regions revealed that immune checkpoint blockade substantially enhanced the trafficking and accumulation of ICOS^+^CD4^+^ and ICOS^+^CD8^+^ T cells within the intratumoral compartment, particularly near the tumor border, whereas vehicle‐treated tumors showed minimal infiltration by resting or activated T cells (Figure 7C and Figure S6D).

To quantitatively assess immune spatial organization, we performed a T cell‐centric neighborhood analysis measuring the mean distance between activated T cells in therapy‐treated mice compared with vehicle controls. Specifically, the mean inter‐T cell distance was 29.8 µm in mice that received combination immune checkpoint blockade, compared with 453.3 µm in vehicle‐treated mice (Figure 7D and Figure S6E). This marked reduction in inter‐T cell distance indicates dense clustering of activated T cells within the TME, suggesting localized immune activation and coordinated immune engagement with TAMCs following therapy.

## 3 Discussion

Metastatic dissemination to distant organs—and particularly to the brain—remains the most serious obstacle in the treatment of melanoma. Melanoma cells harbor intrinsic genomic and phenotypic adaptations that facilitate the metastatic cascade, while the unique biology of MBM impedes effective drug penetration and restricts effector T cell access to intraparenchymal lesions. Due to these challenges, local therapies such as surgical resection, whole‐brain radiation therapy, and stereotactic radiosurgery have historically been the pillars of MBM management, providing regional disease control but exerting limited impact on systemic immunity or long‐term survival. Over the past decade, however, the advent of ICI and targeted therapies has fundamentally reshaped the therapeutic landscape for MBM, enabling durable intracranial responses in a subset of patients and highlighting the central role of immune‐mediated tumor control in the brain.

Despite these advances, accurately monitoring therapeutic responses and predicting clinical outcomes after cancer immunotherapy remains challenging due to the complex, heterogeneous, and highly dynamic spatiotemporal nature of immune responses, particularly within the central nervous system. Contrast‐enhanced MRI remains the clinical gold standard across all phases of brain tumor management, including diagnosis, therapy planning, response assessment, and detection of recurrence, but it primarily reports anatomic changes and lacks specificity for immune cell infiltration or functional immune activation, which are key determinants of immunotherapy success or failure. The ability to detect early immune engagement—or lack thereof—is therefore critically important, as early identification of nonresponding patients could enable timely therapeutic adjustment and avoid prolonged exposure to ineffective treatments. This unmet need has led to increasing interest in molecular imaging strategies capable of detecting immune dynamics directly. In this context, immunoPET imaging holds significant promise for noninvasive, whole‐body visualization of immune processes with high molecular specificity. By directly interrogating immune cell activation rather than static measures of tumor burden, immunoPET approaches have the potential to provide earlier and more biologically informative readouts of therapeutic efficacy, guide treatment adaptation, and improve patient stratification in diseases like MBM, where early immune engagement is both difficult to assess and critical to clinical success.

Motivated by this clinical need for a functionally relevant, immune cell‐specific imaging biomarker, we sought to validate ICOS as a marker of activated T cells following combination immune checkpoint inhibition in a murine model of MBM. Clinically, ICOS expression has been associated with therapy‐engaged effector T cells in patients receiving ICIs and has emerged as a pharmacodynamic indicator of effective immune modulation, making it particularly relevant in the setting of brain metastases where direct tissue sampling is limited [26, 38]. Prior work by Taggart et al. [31] demonstrated that PD‐1/CTLA‐4 blockade in murine MBM primarily enhances the trafficking of CD8^+^ T cells into the brain rather than driving local intratumoral activation or expansion. Extending these findings, our ICOS‐immunoPET data demonstrated that the T cells recruited to the intracranial compartment after checkpoint blockade are functionally engaged, as evidenced by robust ICOS expression on both CD4^+^ and CD8^+^ T cell subsets. Using complementary transcriptomic, flow cytometric, spatial proteomic, and in vivo imaging approaches, we showed that ICOS expression is strongly induced on activated T cells that traffic into MBM following immunotherapy. Importantly, ICOS upregulation was observed not only within intracranial tumors but also in peripheral tumors and immune compartments, indicating that ICOS reflects a dynamic, systemic program of T cell activation rather than a purely tumor‐localized phenomenon.

Our single‐cell spatial proteomics dataset provided a unique opportunity to interrogate how therapy‐induced immune trafficking reshapes the cellular and spatial organization of the MBM immune microenvironment. Prior studies by Smalley et al. and Sun et al. [30, 39] have shown that no‐treatment MBM are typically dominated by tumor cells and immunosuppressive myeloid populations, with very few infiltrating lymphocytes, whereas immune checkpoint blockade promotes increased immune infiltration and diversification of lymphocyte states. Consistent with these observations, we found that immune checkpoint blockade in our murine MBM model was accompanied by a marked expansion and diversification of infiltrating immune cells relative to vehicle‐treated controls. Importantly, our spatial multi‐omics analyses revealed that this increase was not merely quantitative: activated T cells exhibited significantly increased density and spatial proximity within intracranial tumor regions following combination PD‐1/CTLA‐4 blockade, reflecting coordinated immune engagement rather than diffuse or stochastic infiltration. Emerging evidence indicates that spatial immune organization—particularly increased clustering and proximity of CD8^+^ T cells within the tumor core—is a critical determinant of effective antitumor immunity and a strong predictor of immunotherapy response, often outperforming bulk measures of immune cell abundance [40]. In parallel, our reanalysis of scRNA‐seq data from human MBM samples demonstrated that immune checkpoint blockade induces a coordinated shift toward a costimulated, activated T cell state characterized by robust upregulation of ICOS, alongside strengthened immune interaction networks across lymphoid and myeloid populations. These human data closely mirror our preclinical findings and collectively support ICOS as a biologically relevant and promising biomarker for clinical imaging of immunotherapy‐induced T cell activation in the brain.

Several immune‐targeted PET tracers have been developed to noninvasively interrogate antitumor immune responses, but most report immune cell presence or downstream effector activity rather than directly capturing early T cell activation state. CD8‐targeted immunoPET agents, including antibody‐based tracers and emerging fragment‐based approaches such as the ^18^F‐labeled CD8 tracer ^18^F‐GEH200521, are currently in clinical trials and have been used across multiple solid tumor contexts to visualize tumor‐infiltrating CD8^+^ lymphocytes and assess spatial heterogeneity following immune checkpoint blockade [41, 42, 43]. However, because CD8 is expressed by resting, activated, exhausted, and bystander cytotoxic T cells, these tracers cannot distinguish therapy‐engaged, functionally active T cells from nonproductive or quiescent populations, limiting their ability to predict therapeutic efficacy. While metabolic immune tracers such as ^18^F‐F‐AraG offer a complementary strategy by exploiting nucleoside salvage pathways enriched in activated lymphocytes [44], their uptake in other activated or proliferating immune cells and inflamed tissues constrains specificity of the signal for T cells and can complicate signal interpretation, particularly in the brain. More recently, granzyme B‐targeted PET tracers have been developed to image cytotoxic effector function by detecting a protease released by activated CD8^+^ T cells and natural killer cells during immune responses, with early clinical studies (e.g., NCT05000372) demonstrating promise in identifying ongoing immune‐mediated tumor killing [45]. Nonetheless, granzyme B imaging primarily reflects downstream effector activity, which may represent a relatively late phase of the immune response. As such, it may not fully capture earlier immune activation states that precede effector function and could be important for understanding treatment dynamics and predicting longer‐term therapeutic outcomes following immune checkpoint inhibition.

Taken together, these approaches highlight the growing capability of immunoPET to interrogate antitumor immunity, while also underscoring a central limitation of many existing tracers that capture immune cell abundance or terminal effector function rather than the activation state of therapy‐engaged T cells. In this context, activation‐associated markers such as ICOS, OX40, and 4‐1BB represent a biologically distinct and potentially more informative class of imaging targets. Notably, prior studies have demonstrated that ICOS upregulation correlates with tumor response earlier than changes in bulk CD4^+^ or CD8^+^ T cell subsets [25], suggesting that imaging T cell activation may provide earlier and more actionable insights than phenotype‐based measures of immune infiltration. Therefore, systemic evaluation of functional T cell markers is critical to identify immunoPET strategies that provide a functionally proximal and biologically specific readout of immune engagement, ultimately improving response assessment and therapeutic decision‐making in MBM.

Building on this rationale, our study demonstrates the feasibility of ICOS‐immunoPET as a clinically relevant strategy for noninvasive tracking of activated T cells after immunotherapy, with direct applicability to monitoring immune engagement in MBM. Several important limitations still merit consideration. First, although ICOS is a robust marker of T cell activation, its expression does not uniformly correlate with improved survival across all cancer types or treatment contexts. As such, ICOS‐immunoPET signal should be interpreted as a pharmacodynamic marker of immune engagement rather than a standalone prognostic biomarker. Second, BBB integrity may have been altered by intracranial tumor implantation in our murine model, potentially enhancing tracer permeability and contributing to nonspecific retention within brain tumor regions. Importantly, this reflects a clinically relevant aspect of melanoma biology, as MBM are highly invasive and known to compromise BBB integrity through both direct vascular invasion and tumor‐driven inflammatory signaling. To mitigate confounding effects of BBB disruption, we perfused all mice after PET imaging and performed gamma counting of dissected tissues in conjunction with high‐resolution autoradiography of thin brain sections. Both techniques minimize blood pool contribution and nonspecific tracer retention. The resulting ex vivo findings, together with flow cytometric analyses demonstrating elevated ICOS expression on infiltrating T cells within brain tissues, support the biological specificity of the observed imaging signal.

In addition, this study employed a well‐characterized intracranial implantation model of MBM, which provides reproducible and consistent brain tumor establishment and enabled rigorous evaluation of ICOS‐immunoPET as a marker of T cell activation within the brain TME following immunotherapy. While this controlled model was well suited for the objectives of the current study, other MBM models, including spontaneous metastasis models, may be valuable for investigating complementary biological questions related to metastatic dissemination, brain colonization, and disease progression. Another potential limitation of this work is the use of a full‐length antibody as the imaging vector, which is associated with prolonged circulation time, higher nonspecific background signal in highly vascularized organs such as the heart, liver, and spleen, and potential Fc‐mediated binding to other immune cells. Such limitations could be circumvented through alternative vector formats, including antibody fragments or engineered protein scaffolds with reduced size, shorter serum half‐lives and compatibility with short‐lived isotopes, and more favorable pharmacokinetics, which could improve target‐to‐background contrast and better suit clinical imaging applications. Moreover, while the current study focused on imaging at a defined post‐treatment time point, the noninvasive nature of ICOS‐immunoPET offers the opportunity for longitudinal assessment of immune responses throughout therapy. Future studies incorporating serial imaging at baseline, mid‐treatment, and post‐treatment—particularly in the context of novel immunotherapies or combination treatment strategies—could provide important insight into the kinetics of immune activation and therapeutic response. Such studies may be facilitated by the development of smaller ICOS‐targeting vectors compatible with shorter‐lived radionuclides, enabling more practical serial imaging while preserving sensitivity to dynamic changes in T cell activation. Longitudinal approaches could build upon the findings presented here by providing a more comprehensive understanding of the temporal evolution of T cell activation and therapeutic response, further expanding the translational utility of ICOS‐immunoPET for immunotherapy monitoring.

In conclusion, ICOS‐immunoPET is a specific and functionally informative imaging strategy for visualizing therapy‐mediated T cell activation in MBM. By directly reporting immune activation rather than static tumor burden or immune cell abundance, this class of functional imaging probes has the potential to enable earlier prediction of immunotherapy response, inform selection of single‐agent and combination immunotherapies, and facilitate the development and optimization of new‐generation immunotherapeutic strategies. More broadly, ICOS‐immunoPET could ultimately serve as a noninvasive imaging endpoint for evaluating immunotherapies in both clinical practice and clinical trials, providing timely and accurate readouts of immune activation and guiding strategies to improve outcomes for patients with brain metastases.

## 4 Experimental Section

### 4.1 Animals

Female 6‐to‐8‐week‐old C57BL/6J mice (Jackson Laboratory strain 000664) were housed in a temperature‐controlled environment under a 12‐h light/dark schedule with unrestricted access to food and water. Treatment groups were randomly assigned by cage. Animal numbers for each study and analysis are provided in Table S4. Sample sizes varied between experiments and treatment arms due to logistical constraints associated with conducting multiple independent experimental cohorts. All animal procedures complied with the Animal Welfare Act and Stanford University institutional guidelines and were approved by the Stanford University Institutional Animal Care and Use Committee (IACUC) under protocol 32577 (institutional assurance number A3213‐01). Stanford University's animal care program is accredited by the Association for the Assessment and Accreditation of Laboratory Animal Care International (AAALAC International).

### 4.2 Cell Culture

Murine B16F1‐luc was procured from American Type Culture Collection (ATCC, Manassas, VA) and cultured in Dulbecco's Modified Eagle Medium (DMEM; Gibco, Billings, MT) with 10% fetal bovine serum (FBS; Sigma‐Aldrich, St. Louis, MO) and 1% penicillin‐streptomycin (Sigma–Aldrich). All cells were kept in a 37°C humidified incubator with 5% CO_2_. In preparation for tumor implantation, cells were trypsinized using 0.05% trypsin‐EDTA (Gibco) and washed in phosphate‐buffered saline (PBS, Gibco). Viability and quantity were assessed using an automated cell counter and 0.4% Trypan Blue (Gibco) staining. 5 × 10^3^ cells were injected for intracranial tumor implantation and 0.25 × 10^6^ cells for extracranial tumor implantation.

### 4.3 Tumor Implantation

Detailed methodology for intracranial tumor implantation has been previously described [46]. In brief, mice were anesthetized via intraperitoneal (i.p.) injection with ketamine‐xylazine solution. The surgical area was sanitized, and the surface of the skull was exposed via a small midline incision. A left‐sided burr hole was drilled at the following coordinates: 2 mm anterior and 2 mm lateral to lambda. 5 × 10^3^ B16F1‐luc cells in 2 µL PBS were stereotactically implanted into the left striatum, 3 mm deep to the cortical surface. Sham‐operated mice received PBS only. For extracranial tumor implantation, 0.25 × 10^6^ B16F1‐luc cells suspended in 100 µL sterile PBS were mixed 1:1 with Matrigel (BD Biosciences, Franklin Lakes, NJ) and subcutaneously injected into the right flank of female C57BL/6J mice. Tumor growth was monitored daily starting on day 7 post‐extracranial tumor implantation using an electronic caliper. Tumor volume was calculated using the following equation: volume = (π/6) x height x width x length.

### 4.4 Drug Treatments

Mice were randomly assigned to receive either ICI therapy or vehicle control. All therapies were administered intraperitoneally on days 5, 7, and 9 following intracranial tumor implantation. The ICI regimen consisted of anti‐PD‐1 (100 µg in 100 µL; clone BE0146) and anti‐CTLA‐4 (100 µg in 100 µL; clone BE0164) mAbs (Bio X Cell, Lebanon, NH) administered concurrently. Vehicle control mice received an equivalent total injection volume of sterile saline (200 µL, i.p.).

### 4.5 Bioluminescent Imaging

On day 5 post‐tumor inoculation, tumor‐bearing mice were injected with luciferin (150 µL, 15 mg/mL in PBS, i.p.) and subsequently imaged on an In Vivo Imaging System (IVIS; PerkinElmer, Waltham, MA). Bioluminescent signal acquisition and analysis were performed using Living Image 4.1 software. Mice were randomized into treatment and vehicle groups based on the intracranial bioluminescence signals to ensure equal distribution of tumor burden across groups.

### 4.6 Flow Cytometry

On day 11, mice were euthanized, and brains, spleens, and livers were harvested in complete Roswell Park Memorial Institute Medium (RPMI) after PBS perfusion. Brains and spleens were mechanically dissociated by passing the tissue through a 70 µm strainer (Corning) using the plunger of a 3 mL syringe to produce a single‐cell suspension. Liver tissues were enzymatically digested and subsequently filtered through a 70‐µm cell strainer to obtain single‐cell suspensions. Brain single‐cell suspensions were centrifuged through a continuous 30% Percoll gradient to remove myelin and other cell debris. Cells were resuspended in FACS buffer (2% FBS in PBS) and stained with Zombie NIR live/dead (BioLegend, 423105), AF488 CD45 (BioLegend, 160305), BUV395 CD3 (BD Biosciences, 569614), RB780 CD8 (BD Biosciences, 568693), RY610 CD4 (BD Biosciences, 571147), APC ICOS (BioLegend, 107711). Samples were washed, paraformaldehyde (PFA)‐fixed (2% PFA, 20 min, room temperature, and acquired on the Cytek Aurora (Cytek Biosciences, Fremont, CA). FlowJo software (v10, BD Biosciences) was used for the analysis and depiction of the gating strategy.

### 4.7 RNA Extraction, cDNA Synthesis, and Quantitative RT‐PCR

Whole brains were frozen in TRIzol (Thermo Fisher Scientific, Waltham, MA) immediately after gamma counting. RNA isolation was performed according to the manufacturer's recommended protocol (Thermo Fisher Scientific). Complementary DNA was synthesized using the RT^2^ First Strand Kit (Qiagen, Redwood City, CA). Gene expression analysis was conducted using primers targeting *Icos* (Qiagen), with *Gapdh* serving as the reference housekeeping gene. qPCR was performed on an Applied Biosystems QuantStudio 6 system (Thermo Fisher Scientific). All samples were analyzed in triplicate technical replicates, and fold change for each gene was calculated by deriving 2^−ddC^_T_.

### 4.8 Bioconjugation and ^89^Zr Radiolabeling of Monoclonal Antibodies

^89^Zr radiolabeled ICOS antibody (rat clone 7E.17G9, Bio X Cell) was prepared as previously described [25]. Briefly, 1 mg of antibody was diluted to 1 mg/mL in PBS, and the pH was adjusted to 8.8 to 9.0 prior to addition of deferoxamine‐isothiocyanate (DFO‐SCN) chelate (Macrocyclics) dissolved in dimethyl sulfoxide (DMSO; Thermofisher). The bioconjugation reaction was allowed to proceed for 1 h at 37°C, after which the DFO‐modified mAb was washed using a 2 mL Vivaspin filter with a 50 kDa cutoff (Sartorius) to remove unbound chelate. Matrix‐assisted laser desorption/ionization (MALDI)‐mass spectrometry was conducted on the bioconjugates to determine final chelate‐to‐mAb ratios, and final protein concentrations were determined using a Thermo Scientific NanoDrop UV–vis spectrophotometer. For radiolabeling, ∼1 mCi ^89^Zr oxalate (3D Imaging), adjusted to pH 7.1 to 7.8 using 1 M Na_2_CO_3,_ was incubated with the DFO‐ICOS mAb for 70 min at 37°C with gentle agitation. Free ^89^Zr oxalate was removed, and ^89^Zr‐DFO‐ICOS mAb was purified using 7K MW cutoff zeba spin desalting column (Thermofisher) and centrifuged for 1 min at 1000 g. Final radiochemical purity of >99% of ^89^Zr‐DFO‐ICOS mAb was determined using instant thin‐layer chromatography using chromatography strips (Biodex).

### 4.9 In Vivo PET/CT/MR Imaging Studies

Mice were anesthetized with isoflurane gas and intravenously injected with ^89^Zr‐DFO‐ICOS mAb (50 µCi; 0.5 µCi/µL, mass dose injected; 7 µg) on day 11 post‐intracranial tumor implantation. At 72 h post‐tracer injection, 20‐min static PET scans were acquired using the GNEXT scanner (Sofie Biosciences, Dulles, VA) in list mode format. CT images were acquired after each PET scan to provide an anatomical reference frame in addition to scatter and attenuation correction for PET data. Scanner calibration factors were determined using ^89^Zr standards to ensure accurate PET quantification. PET images were reconstructed using an OSEM3D/MAP algorithm (24 subsets, 3 iterations) with a matrix size of 240 × 240 × 191. Following PET/CT imaging, mice underwent head‐and‐neck MRI using an actively shielded Bruker 7T horizontal bore scanner (Bruker Corp, Billerica, MA), with International Electric Co. (IECO) gradient drivers, a 120 mm ID shielded gradient insert (600 mT/m, 1,000 T/m/s), AVANCE III electronics, 8‐channel multi‐coil RF and multinuclear capabilities and volume RF coils, and the supporting ParaVision 360 V3.7 platform. T2‐coronal and axial images were acquired with the following parameters: echo time: 33 ms; repetition time: 2,500 ms; 2 averages; 17 slices with a thickness 0.5 mm and a voxel size of 0.25 mm × 0.1556 mm × 0.5 mm.

### 4.10 PET Image Analysis

Regions of interest (ROIs) were analyzed using Inveon Research Workplace (Siemens Healthineers, Erlangen, Germany). Briefly, CT images (for peripheral organ analysis) or T2‐weighted MRI (for brain analysis) were co‐registered with PET images to provide anatomical reference for ROI delineation. For brain tumor analyses, ROI was guided by MRI and further verified using corresponding H&E‐stained brain sections. PET data were normalized to injected dose and expressed as %ID/g.

### 4.11 Ex Vivo Gamma Counting and Autoradiography

Immediately after PET, cardiac puncture was performed under anesthesia. Mice were subsequently perfused with 20 mL of PBS and tissues of interest (brain, liver, spleen, bone, bone marrow, muscle, extracranial tumor, and tail) were individually harvested, weighed wet, and gamma‐counted (Hidex automatic gamma counter, Hidex, Turku, Finland). Biodistribution results were decay‐corrected and calibrated using ^89^Zr standards (∼3 µCi/standard) and expressed as %ID/g based on the wet weight of each dissected organ. Brains were further analyzed via digital autoradiography. Briefly, whole brains were frozen in optimal cutting temperature (OCT) compound on dry ice following gamma counting, cryosectioned into thin (40 µm) slices, and exposed to digital phosphorus films for five half‐lives. Films were scanned using a Typhoon phosphorimager (Amersham Biosciences, Amersham, United Kingdom). Autoradiography images were analyzed in ImageJ by manually delineating ROIs over tumor regions to determine mean signal intensity. Dose‐corrected autoradiography values were calculated by dividing the mean ROI signal intensity by the decay‐corrected radioactivity present in the corresponding tissue section at the time of film exposure. The same brain sections were subsequently stained with hematoxylin and eosin (H and E; Hematoxylin Gills 3, Thermo Fisher Scientific; Eosin‐Y Richard‐Allan Scientific, Thermo Fisher Scientific) for anatomical reference and visualization of tissue morphology and immune cell infiltration.

### 4.12 Automated Hyperplex IF Staining and Imaging on COMET

Automated hyperplex IF staining and imaging were performed on the COMET platform (Lunaphore Technologies, Tolochenaz, Switzerland). Slides underwent 11 cycles of iterative staining and imaging, followed by an elution of the primary and secondary antibodies [35]. In brief, FFPE slides were preprocessed with PT Module (Epredia, Kalamazoo, MI) with Dewax and HIER Buffer H (AR03, Lunaphore Technologies) for 60 min at 102°C. Subsequently, slides were rinsed and stored in a Multistaining Buffer (BU06, Lunaphore Technologies) until use. The 15‐plex protocol template was generated using the COMET Control Software, and reagents were loaded onto the instrument to perform the seqIF protocol. A blocking step before staining of 15 biomarkers was done with the Blocking buffer (BU10, Lunaphore Technologies). Nuclear signal was detected with DAPI (Lunaphore Technologies, code DR100) by dynamic incubation for 2 min. All reagents were diluted in Multistaining Buffer (BU06, Lunaphore Technologies). The elution step lasted 2 min for each cycle and was performed with Elution Buffer (BU07‐L, Lunaphore Technologies) at 37°C. The quenching step lasted for 30 s and was performed with Quenching Buffer (BU08‐L, Lunaphore Technologies). The imaging step was performed with Imaging Buffer (BU09, Lunaphore Technologies). Once the experiment was completed, a raw OME‐TIFF file was generated by the COMET Control software for downstream analysis.

### 4.13 SeqIF Analysis

After image acquisition and preprocessing, nuclei‐based cell segmentation was performed using DAPI staining and a StarDist‐based machine learning model implemented in the HORIZON software [36, 37]. Segmentation outputs were filtered based on cell size, spatial localization, and mean fluorescence intensity across channels to exclude artifacts and poorly segmented objects. For cell phenotyping, fluorescence intensities for each marker were preprocessed by winsorizing the upper 0.01% of values to remove outliers, followed by Z‐score normalization (mean centering and variance scaling). Cells were subsequently classified using a rule‐based binary decision tree constructed from the expression patterns of 13 phenotypic markers (Figure S6). Spatial distance between cell phenotypes was calculated using a k‐nearest neighbors (k‐NN) algorithm, measuring the Euclidean distance between each target cell and its nearest neighboring cell of a specified phenotype. Mean intracellular distances were computed to assess spatial clustering and immune organization within tumor regions. Processed data were exported to Python for downstream analysis and visualization using Pandas and Matplotlib.

### 4.14 Single‐Cell RNA Sequencing Analysis

Single‐cell RNA sequencing data from human MBM were reanalyzed using the publicly available dataset GSE193745 [30]. Clinical metadata—including patient ID, mutation status, treatment history, and treatment group (ICI vs. no‐treatment)—were manually curated from the published study and merged with cell‐level annotations. For the ICI responder cohort, samples were selected from patients who demonstrated clinical benefit and prolonged survival after treatment. Raw count matrices were processed using Scanpy (v1.10). Gene expression was normalized per cell, log‐transformed, and scaled. Highly variable genes were identified using the Seurat v3 method, followed by principal component analysis and construction of a k‐nearest neighbor graph. Unsupervised Leiden clustering was performed separately for no‐treatment and ICI‐treated samples, and clusters were visualized using t‐SNE plots. Cell clusters were annotated based on established lineage‐specific marker genes. Cell‐cell communication was inferred using an expression‐based ligand‐receptor pathway framework encompassing immune and inflammatory signaling axes. Interaction strength between cell types was calculated as the product of mean ligand expression in source populations and mean receptor expression in target populations.

### 4.15 Statistical Analysis

Statistical analyses were performed using Prism (version 10, GraphPad Software, San Diego, CA). Statistical analyses were performed using Mann‐Whitney tests, one‐way analysis of variance (ANOVA) with multiple comparisons tests, or Fisher's exact tests as indicated in the figure legends for each figure and table. PET imaging results are pooled from two independent experiments with a total of *n* = 11 vehicle‐treated and *n* = 16 therapy‐treated mice.

## Author Contributions

**Renesmee C. Kuo**: conceptualization, investigation, writing – original draft, visualization, methodology, validation, formal analysis, software. **Pedro Machado Almeida**: investigation. **Josué Ballesteros‐álvarez**: investigation. **Israt S. Alam**: conceptualization, funding acquisition, supervision, writing – review and editing. **Michael Lim**: conceptualization, funding acquisition, supervision, project administration. **Mausam Kalita**: investigation. **Rohit Verma**: methodology, investigation. **Hannes Vogel**: supervision, resources. **Michelle L. James**: conceptualization, funding acquisition, supervision, project administration, writing – review and editing. **Samuel Aubert**: investigation. **Sydney C. Nagy**: investigation.

## Conflicts of Interest

S.A., J.B.A., and P.M.A. are employed by Lunaphore Technologies during the completion of this project. All other authors have declared that no competing interests exist.

## Supporting information




**Supporting File**: advs76803‐sup‐0001‐SuppMat.pdf.

## Data Availability

All data associated with this study are present in the paper and/or the Supplementary Information. The raw data files for the previously published and publicly available scRNA‐seq data are available in the Gene Expression Omnibus database (GSE193745).
