# Supplementary material for: ICOS‐ImmunoPET Monitors T Cell Activation After Combination Immune Checkpoint Blockade in a Mouse Model of Melanoma Brain Metastasis
Source: Adv Sci (Weinh). 2026 Aug 15;13(56):e76803. doi: 10.1002/advs.76803 (PMC13477249; doi:10.1002/advs.76803)
Supplement: Supplementary file 1 [file ADVS-13-e76803-s001.pdf]

ICOS-ImmunoPET Monitors T Cell Activation after Combination Immune Checkpoint Blockade in a Mouse Model of Melanoma Brain Metastasis

Renesmee C. Kuo[1,2], Rohit Verma[3], Sydney C. Nagy[2], Mausam Kalita[2], Samuel Aubert[4], Josué Ballesteros-Álvarez[4], Pedro Machado Almeida[4], Hannes Vogel[5], Israt S. Alam[2]*, Michael Lim[3]*, Michelle L. James[2,6]*

[1]*Department of Electrical Engineering, Stanford University, Stanford, CA, USA*

[2]*Molecular Imaging Program at Stanford, Department of Radiology, Stanford University, Stanford, CA, USA*

[3]*Department of Neurosurgery, Stanford University, Stanford, CA, USA*

[4]*Lunaphore Technologies, Tolochenaz, Switzerland*

[5]*Department of Pathology, Stanford University, Stanford, CA, USA.*

[6]*Department of Neurology and Neurological Sciences, Stanford University, Stanford, CA, USA*

*Corresponding authors:

Michelle L. James, 3172 Porter Drive, Room 273, Palo Alto, CA 94304; E-mail: mljames@stanford.edu

Michael Lim, 1201 Welch Road, MSLS P350, Palo Alto, CA 94305; E-mail: mklim@stanford.edu

Israt S. Alam, 3172 Porter Drive, Room 260, Palo Alto, CA 94304; E-mail: israt@stanford.edu

Supplementary Figures

- **Figure S1:** Flow cytometry gating strategy of a representative mouse
- **Figure S2:** Intracranial tumor volume measured by MRI in vehicle- and therapy-treated MBM mice
- **Figure S3:** Radiolabeling and characterization of ^{89}Zr-DFO-ICOS mAb
- **Figure S4.** Representative ICOS-immunoPET/CT images across multiple tumor sections in naïve and MBM mice
- **Figure S5:** ICOS expression is elevated in the liver of therapy-treated melanoma brain metastasis mice
- **Figure S6:** Spatial proteomic characterization of the brain tumor microenvironment in melanoma brain metastasis mice

Supplementary Tables

- **Table S1:** Final specifications of ^{89}Zr-DFO-ICOS mAb
- **Table S2:** PET analysis and tumor-to-background ratios in melanoma brain metastasis mice
- **Table S3:** Biomarker panel used for multiplexed immunofluorescent imaging
- **Table S4:** Number of mice used in preclinical studies. Results pooled from two independent experiments

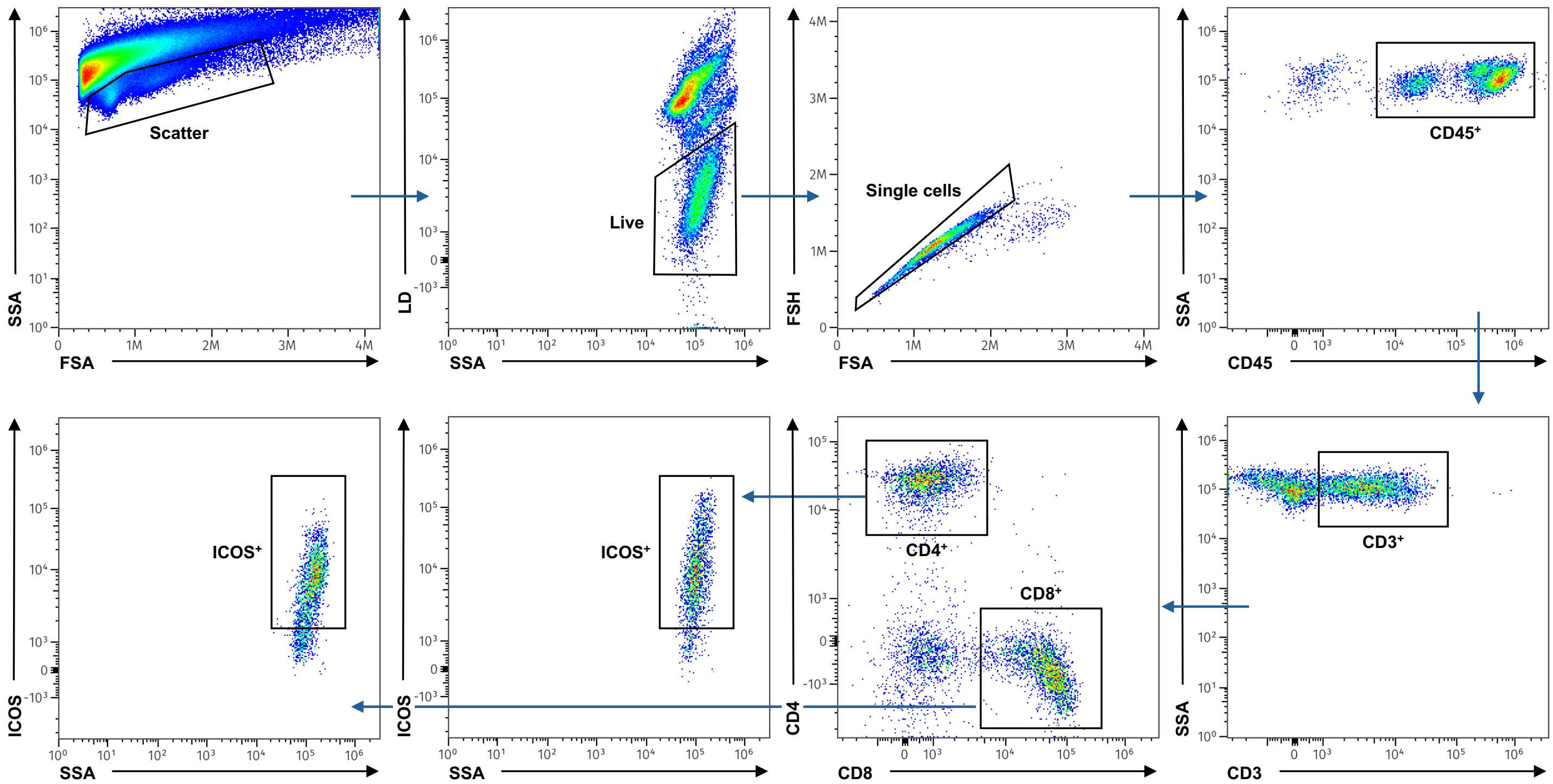

Figure S1. Flow cytometry gating strategy of a representative mouse to differentiate live single cells into activated helper T cells ($CD45^+CD3^+CD4^+ICOS^+$) and activated cytotoxic T cells ($CD45^+CD3^+CD8^+ICOS^+$).

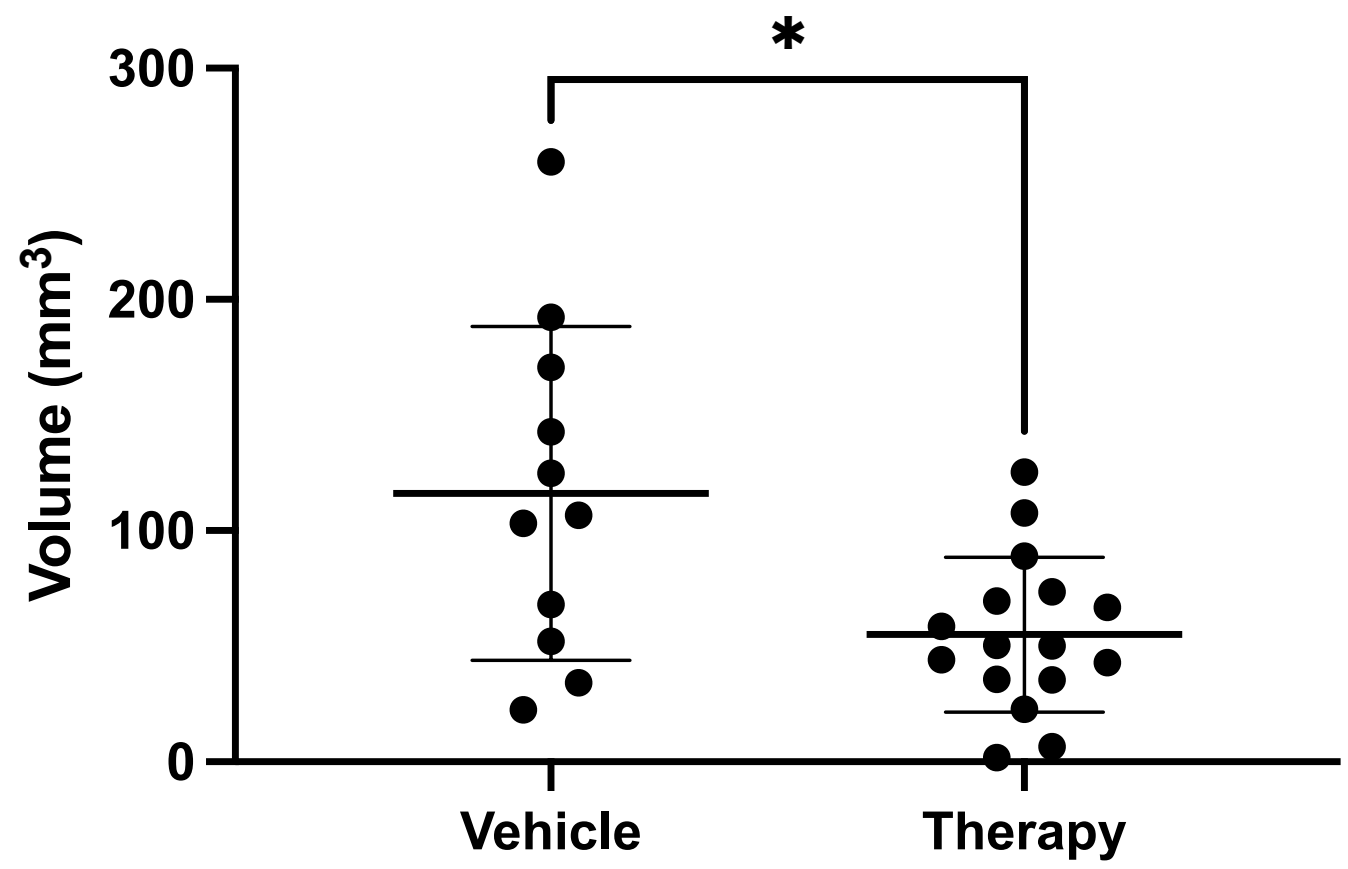

Figure S2. Intracranial tumor volume measured by MRI in vehicle- and therapy-treated MBM mice on day 14 post-intracranial implantation. Statistical analysis was performed using Mann-Whitney test (*denotes direct comparison of groups, *$p<0.05$). Data are mean ± SD.

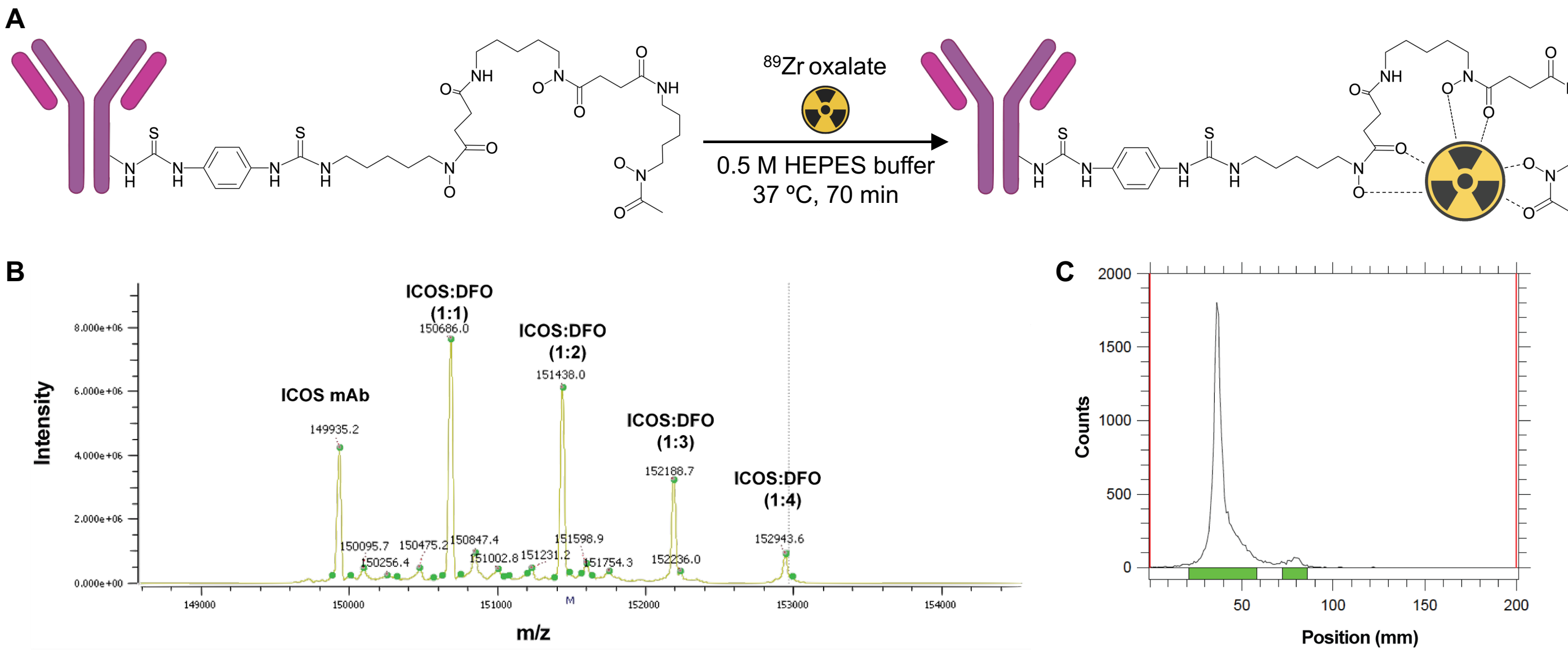

Figure S3. Radiolabeling and characterization of ^{89}Zr-DFO-ICOS mAb. (A) Scheme summarizing the optimized radiolabeling conditions for conjugation of conditions of ^{89}Zr to DFO-modified ICOS mAb. (B) MALDI-mass spectrometry analysis of ICOS mAb before and after DFO conjugation. Peaks corresponding to unconjugated ICOS mAb and ICOS-DFO with increasing chelator-to-antibody ratios (1:1 to 1:4) are indicated. (C) Radio-iTLC results after labeling.

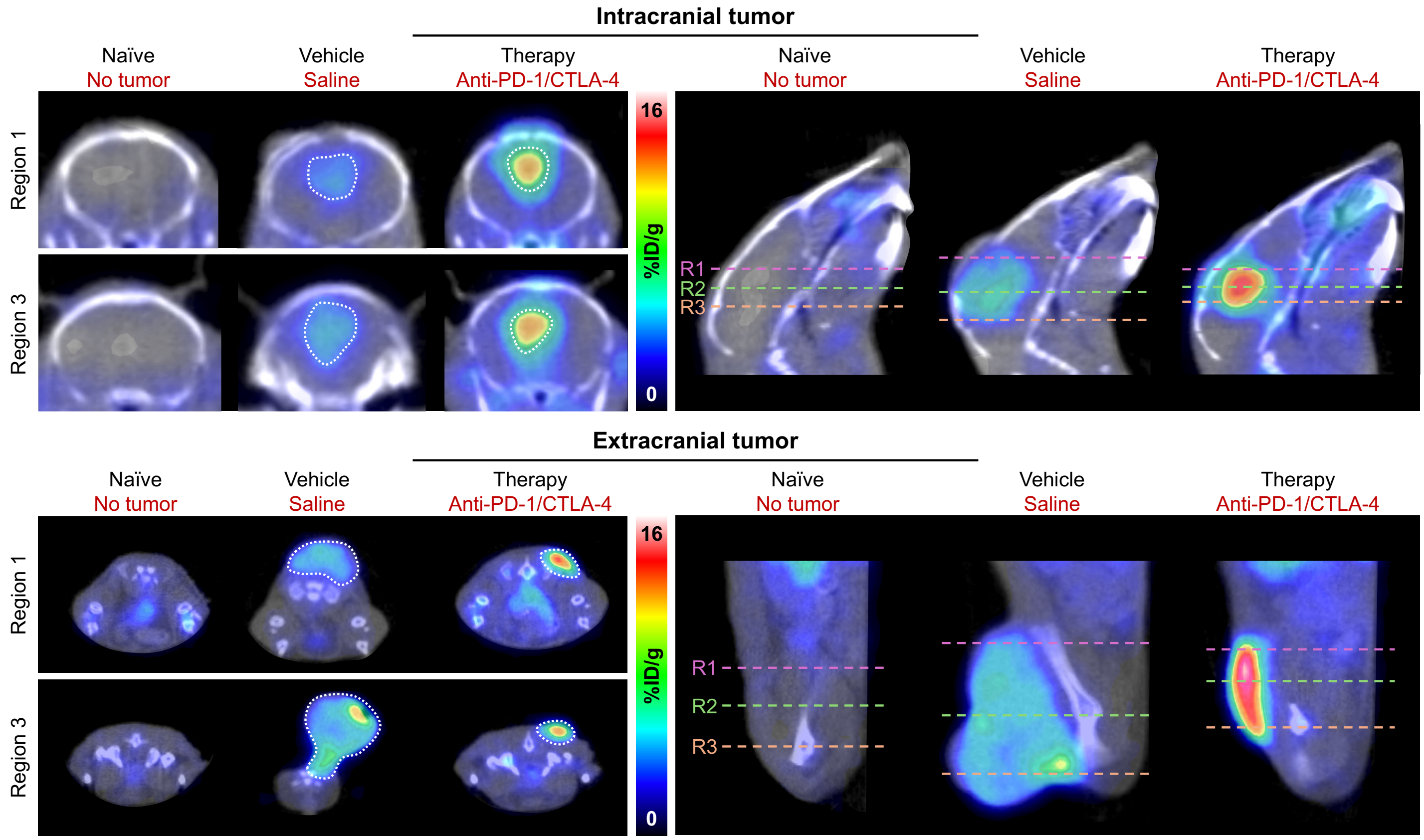

Figure S4. Representative ICOS-immunoPET/CT images across multiple tumor sections in naïve and MBM mice. Representative coronal (left) and sagittal (right) ICOS-immunoPET/CT images of naïve and MBM-bearing mice treated with vehicle or combination anti-PD-1/anti-CTLA-4 therapy, acquired 72 h following ^{89}Zr-DFO-ICOS mAb administration. Images illustrate tracer binding across multiple sections of the intracranial (top) and extracranial (bottom) tumors. Coronal images depict representative anterior (Region 1) and posterior (Region 3) tumor sections, while sagittal images depict the corresponding tumor regions spanning the anterior (R1), central (R2), and posterior (R3) portions of the tumors. Region 2 corresponds to the representative tumor sections shown in Figures 4A-B. White dotted lines indicate tumor boundaries. R1 = region 1; R2 = region 2 (Figure 4A-B); R3 = region 3.

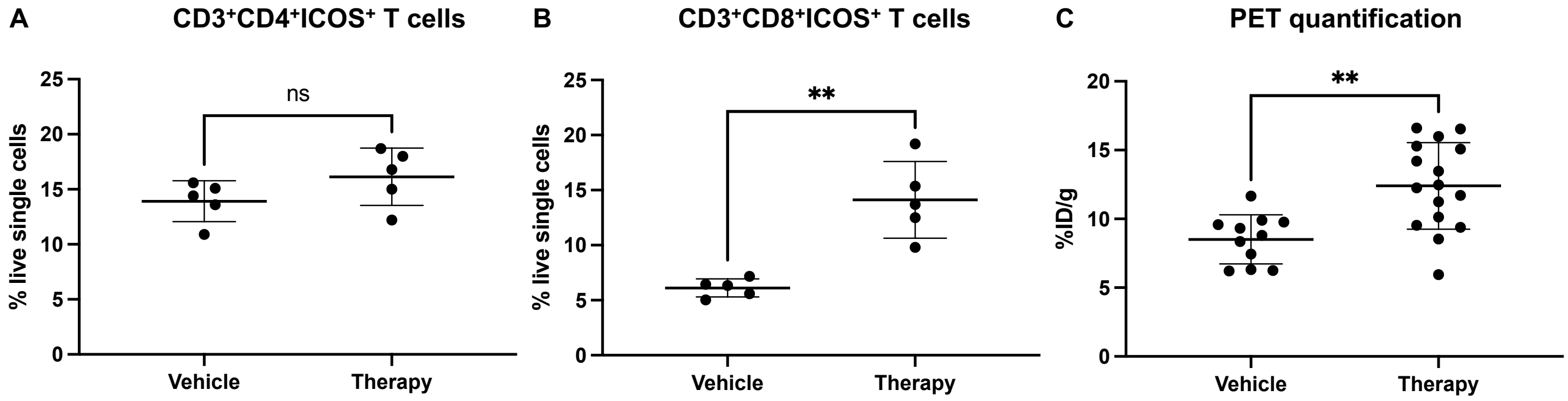

Figure S5. ICOS expression is elevated in the liver of therapy-treated melanoma brain metastasis mice. (A) Flow cytometry quantification of $ICOS^+CD3^+CD4^+$ T cells and (B) $ICOS^+CD3^+CD8^+$ T cells in the liver of vehicle- and therapy-treated mice. (C) PET image quantification of ^{89}Zr-DFO-ICOS mAb signal in the liver. Statistical analysis was performed using Mann-Whitney test. * denotes direct comparison of groups (**p<0.01). Data are mean ± SD.

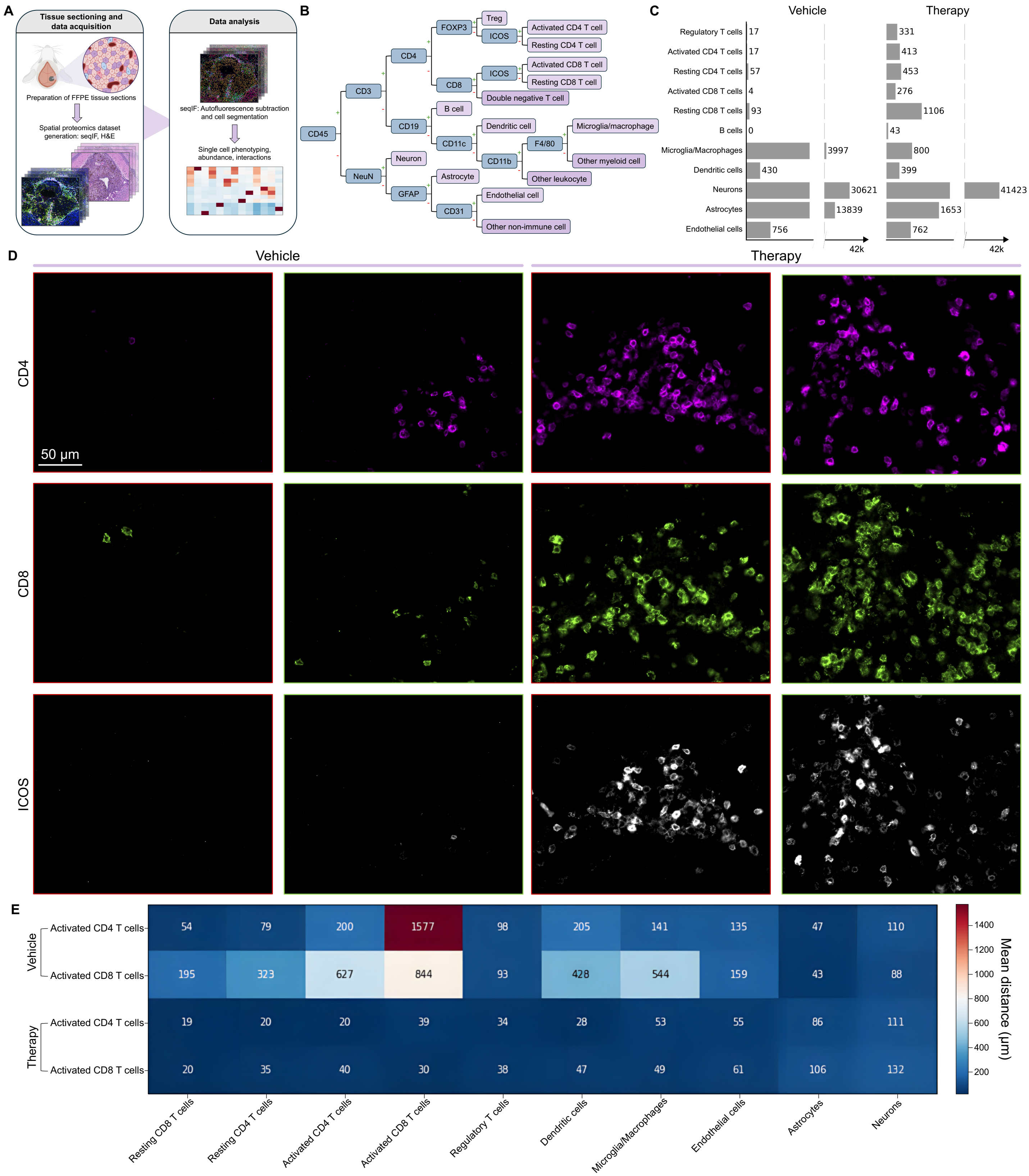

Figure S6. Spatial proteomic characterization of the brain tumor microenvironment in melanoma brain metastasis mice. (A) Overview of the spatial proteomics workflow and analysis pipeline. (B) Rule-based decision tree classifier used to assign cell types to individual cells based on binarized marker expression profiles.

Marker expression was binarized (positive vs. negative) using a previously described automatic thresholding approach (47). (C) Quantification of total cell counts in representative whole brain sections from vehicle- and therapy-treated mice for each phenotypic class. (D) Representative 20x magnification images of whole-brain tissue sections from vehicle- and therapy-treated mice, showing individual immunofluorescence channels for CD4 (pink), CD8 (green), and ICOS (white) corresponding to those presented in Figure 6C. (E) T cell-centric neighborhood analysis depicting the mean distance (μm) between activated $CD4^+$ and activated $CD8^+$ T cells and all other major cell classes across the brain.

Supplementary Table 1: Final specifications of ^{89}Zr-DFO-ICOS mAb.

**Average DFO/ICOS mAb (MS)**	1.59
**Radiochemical purity (iTLC)**	95.89%
**Radiochemical concentration**	14.874 MBq/mL
**Specific activity**	0.308 MBq/μg

Supplementary Table 2: PET analysis and tumor-to-background ratios in melanoma brain metastasis mice (vehicle: n=11; therapy: n=16).

	IC tumor	Healthy brain	Tumor-to-brain ratio	EC tumor	Muscle	Tumor-to-muscle ratio
**Vehicle**	3.28 ± 0.93 %ID/g	0.83 ± 0.32 %ID/g	3.95	4.76 ± 0.68 %ID/g	0.53 ± 0.20 %ID/g	8.98
**Therapy**	4.41 ± 1.03 %ID/g	0.93 ± 0.29 %ID/g	4.73	7.07 ± 1.72 %ID/g	0.56 ± 0.12 %ID/g	12.63

Supplementary Table 3: Biomarker panel used for multiplexed immunofluorescent imaging.

Cycle	Marker	Antibody clone	Supplier	Catalog number	Dilution
**1**	CD3	E4T1B	Cell Signaling Technology	78588S	1:225
**1**	FoxP3	FJK-16s	Invitrogen	14-5773-82	1:30
**2**	CD31	EP3095	Abcam	AB76533	1:200
**2**	CD4	4SM95	Invitrogen	14-9766-82	1:50
**3**	NeuN	EPR12763	Abcam	AB177487	1:1500
**3**	CD8	4SM16	Invitrogen	14-0195-82	1:50
**4**	ICOS	EPR200560	Abcam	AB224644	1:70
**5**	CD19	D4V4B	Cell Signaling Technology	90176S	1:100
**6**	CD11c	D1V9Y	Cell Signaling Technology	97585S	1:130
**7**	CD45	D3F8Q	Cell Signaling Technology	70257S	1:150
**8**	CD11b	EPR1344	Abcam	AB133357	1:5000
**9**	GFAP	Polyclonal	Abcam	AB4674	1:10000
**9**	F4/80	D4C8V	Cell Signaling Technology	30325S	1:450

Table S4: Number of mice used in preclinical studies. Results pooled from two independent experiments.

	Vehicle	Therapy
**Quantitative PCR**	10	7
**Flow cytometry**	10	10
**PET imaging**	11	16
**Biodistribution (gamma counting)**	11	16
**Autoradiography**	5	5
